# Impacts of NO_2_ on Urban Air Quality and Causes of Its High Ambient Levels: Insights from a Relatively Long-Term Data Analysis in a Typical Petrochemical City in the Bohai Bay Region, China

**DOI:** 10.3390/toxics13030208

**Published:** 2025-03-13

**Authors:** Xiaoshuai Gao, Cong An, Yongxin Yan, Yuanyuan Ji, Wei Wei, Likun Xue, Rui Gao, Fanyi Shang, Jidong Li, Luyao Tan, Hong Li

**Affiliations:** 1 State Key Laboratory of Environmental Criteria and Risk Assessment, Chinese Research Academy of Environmental Sciences, Beijing 100012, China; s202265286@emails.bjut.edu.cn (X.G.); yyx_in@163.com (Y.Y.); ji.yuanyuan@craes.org.cn (Y.J.); gaorui@craes.org.cn (R.G.); 2 Department of Environmental Science and Engineering, Beijing University of Technology, Beijing 100124, China; weiwei@bjut.edu.cn; 3 Shanghai Key Laboratory of Atmospheric Particle Pollution and Prevention, Department of Environmental Science and Engineering, Fudan University, Shanghai 200438, China; ancong_22@163.com; 4 Environment Research Institute, Shandong University, Qingdao 266237, China; xuelikun@sdu.edu.cn; 5 Dongying Municipal Ecology and Environment Bureau, Dongying 257000, China; dyhbsfy@126.com (F.S.); shbjzlb@dy.shandong.cn (J.L.); 6 Ltd. of Shandong Environmental Protection Industry Corp., Jinan 250061, China; tanluyyy@163.com

**Keywords:** NO_2_ pollution characteristics, impacts on air quality, pollution meteorological conditions, causes for high NO_2_ values, Bohai Bay region of China

## Abstract

The ambient levels of NO_2_ in urban areas in China in recent years have generally shown a downward trend, but high NO_2_ concentrations still exist under certain conditions, and the causes for such phenomenon and its impact on air quality remain unclear. Taking Dongying, a typical petrochemical city in the Bohai Bay of China, as an example, this paper analyzed the influence of NO_2_ on urban air quality and investigated the causes for the formation of NO_2_ with high concentrations. The results indicated that higher daily NO_2_ concentrations (>40 μg/m^3^) mainly occurred during January-April and September-December each year, and higher hourly NO_2_ concentrations mainly occurred during the nighttime and morning rush hour in Dongying from 2017 to 2023. With the increase in daily NO_2_ concentrations, the daily air pollution levels showed a general increasing trend from 2017 to 2023. The occurrence of high NO_2_ values in Dongying was affected by the combination of unfavorable meteorological conditions, local emissions and regional transports, and localized atmospheric chemical generation. High-pressure and uniform-pressure weather patterns in 2017–2022, along with land–sea breeze circulation in 2022, contribute to high NO_2_ concentrations in Dongying. Boundary layer heights (BLH) in spring (−0.43) and winter (−0.36), wind direction in summer (0.21), and temperature in autumn (−0.46) are the primary meteorological factors driving NO_2_-HH (High hourly NO_2_ values), while BLH (−0.47) is the main cause for NO_2_-HD (High daily NO_2_ values). The titration reaction between NO with O_3_ is the main cause for NO_2_-HH in spring, summer and autumn, and photochemical reactions of aromatics have a significant influence on NO_2_-HD. NOx emissions from the thermal power and petrochemical industry in Dongying and air pollution transports from western and southwestern Shandong Province (throughout the year) and from the Bohai Sea (during spring and summer) had serious adverse impact on high NO_2_ values in 2022. The results of the study could help to provide a scientific basis for the control of NO_2_ and the continuous improvement of air quality in Dongying and similar petrochemical cities.

## 1. Introduction

Atmospheric nitrogen oxides (NOx) include compounds such as nitrous oxide (N_2_O), nitric oxide (NO), nitrogen dioxide (NO_2_), dinitrogen trioxide (N_2_O_3_), and dinitrogen tetroxide (N_2_O_4_). Among these, NO_2_ is one of the more stable nitrogen oxides, with higher concentration and longer persistence, playing a significant role in atmospheric chemical reactions) [1]. The formation of NO_2_ in the atmosphere is primarily related to the conversion of NO. During the day, the photochemical chain reactions involving volatile organic compounds (VOCs) are key to NO_2_ generation, while at night, the reaction of ozone (O_3_) with NO is the main pathway for NO_2_ generation (Figure S1). The photochemical reactions between NO_2_ and VOCs during the day are the only chemical source of O_3_ [2]. The reaction between NO_2_ and hydroxyl radicals (OH) is the primary source of gaseous nitric acid (HNO_3_), and the reactions between NO_2_ and alkoxy radicals (RO) are the main sources of organic nitrate compounds in the atmosphere. NO_2_ reacts with O_3_ to form nitrate radical (NO_3_) at night when NO concentration is low. NO_3_ can react with VOCs or with NO_2_ to form dinitrogen pentoxide (N_2_O_5_), which then reacts with water or aerosols to produce nitric acid (HNO_3_) or nitrate salts (NO_3_^−^), ultimately leading to the net removal of NO_2_ [3]. NO_2_ and its related products, such as O_3_ and organic nitrate compounds, are key components of photochemical smog, while HNO_3_ or NO_3_^−^ are major contributors to acid rain. These pollutants pose potential risks to human health and the eco-environment, they could damage human cardiovascular and pulmonary functions, and other organisms in ecosystems; In addition, photochemical smog could irritate the eye membrane of humans, reduce atmospheric visibility, and contribute to global warming, and acid rain could lead to soil acidification and building corrosion [4,5].

Therefore, studying the pollution characteristics of NO_2_, its impact on air quality, and the causes of high NO_2_ concentrations is crucial for further understanding atmospheric chemical mechanisms, continuously improving air quality, and reducing its adverse effects on human health and the environment.

The distribution pattern of NO_2_ in the atmosphere results from the combined effects of pollution sources, meteorological conditions, and chemical generation. NO_2_ sources are mainly divided into natural and anthropogenic sources [6]. Natural sources include lightning, soil emissions, and ammonia oxidation, while anthropogenic sources include industrial facilities and mobile emissions. Ambient NO_2_ concentrations are greatly influenced by anthropogenic sources [7,8], which is the primary causes for the “higher in the east, lower in the west” distribution pattern observed in China over the years [9]. The average NO_2_ concentration in autumn and winter are relatively higher than in spring and summer [10]. The seasonal differences in anthropogenic NOx emissions and meteorological conditions are important factors contributing to the seasonal variation in NO_2_ concentration [11]. The diurnal variation of NO_2_ concentration usually shows a “two peaks and one valley” pattern. The morning peak can be attributed to motor vehicle emissions, while the evening peak may be related to unfavorable weather conditions and the conversion of NO with high concentrations during the evening rush period [12]. Additionally, NO_2_ exhibits a certain transport capacity [13]. Cheng et al. [14] found that implementing an odd-even license plate policy in the surrounding areas of Beijing significantly reduced NO_2_ concentration in the city. Wang et al. [15] found that controlling NOx emissions in upwind cities is crucial for mitigating O_3_ pollution in specific cities. Relevant studies have found that meteorological conditions, especially wind speeds (WS) and wind direction (WD), often affect the regional transports of NO_2_, leading to high urban NO_2_ concentrations [16,17]. High NO_2_ concentrations at low WS are influenced by nearby pollution sources, while at high WS are influenced by long-distance transport. Xiao et al. [18] found that under weak large-scale meteorological conditions, local circulations related to terrain can also affect pollutant concentrations. The land–sea breeze characteristics in the Bohai Bay region are distinct [19]. During pollution events, offshore land winds can transport pollutants from Shandong Province to the Bohai Sea, while onshore sea winds can create a convergence zone, inducing upward air currents that cause pollutants to cycle over the bay, further deteriorating air quality in coastal areas [20]. Therefore, to fully understand the pollution characteristics of NO_2_ and determine the causes for the formation of NO_2_ with high concentrations, it is necessary to investigate from multiple perspectives.

Over the past 20 years, NOx emissions in China have initially increased and then decreased annually, peaking in 2012. Although NOx emissions in China have decreased, the emission structure in many regions has shifted to being dominated by traffic sources [21,22,23]. However, the overall NOx emissions remain above ten million tons, leading to persistently high ambient NO_2_ concentrations in China [24]. The China Ecological Environment Status Bulletin shows that after a sharp decline in 2014 (34.8 µg/m^3^), NO_2_ concentrations in China’s urban areas fluctuated from 2015 to 2019 (27.5–28.4 µg/m^3^), continued to decline from 2019 (27 µg/m^3^) to 2022 (21 µg/m^3^) but rebounded in 2023 (22 µg/m^3^) with a year-on-year increase of 4.8% [25].Currently, high NO_2_ values in China are concentrated in economically developed and densely populated regions of North China, East China, and Central China [26], primarily in autumn and winter. For example, the monthly average NO_2_ concentration in Jinan, Shandong Province, exceeded 60 μg/m^3^ in January from 2015 to 2021 [27]. Some cities also experienced peak concentration at night during the summer. In 2018, four prefecture-level cities in Shanxi Province experienced pollution peaks during summer, with the average hourly NO_2_ concentration in Lvliang exceeding 80 μg/m^3^ at night [28]. Besides O_3_, PM_2_._5_, and PM_10_, NO_2_ remains one of the primary pollutants on days with air quality exceedance in urban areas of China [25,29]. For example, the number of days with NO_2_ as the primary pollutant and the number of NO_2_ exceedance days increased annually from 2018 to 2020 in Chuzhou, Anhui Province, in the Yangtze River Delta region. In 2020, the proportion of days with NO_2_ as the primary pollutant reached 5.2–9.0% in coastal cities like Qingdao, Binzhou, Rizhao, and Dongying in Shandong Province [30,31].

Dongying City, located in the northwest of Shandong Province and bordered by Bohai Bay to the north and Laizhou Bay to the east, is one of the representative cities in the Bohai Bay of China. Dongying has a developed petrochemical industry. Transportation primarily relies on road traffic, with extensive motor vehicle use and a high number of trucks in urban areas, leading to substantial NO_X_ and VOC emissions in Dongying [32], resulting in various environmental issues, such as increased photochemical pollution and frequent smoggy weather [33]. Previous studies on air pollution in Dongying have mainly focused on aerosols, particulate matter, O_3_, and their precursors [31,32]. Research on NO_2_ has primarily concentrated on pollution characteristics [8,15], with few comprehensive analyses of the impacts and causes of high NO_2_ values. This study analyzed the temporal and spatial distribution characteristics of NO_2_ concentrations in Dongying based on atmospheric pollutant and meteorological data from 2017 to 2023. The impact of NO_2_ on air quality and of high NO_2_ values in 2022 on O_3_, PM_2_._5_, PM_2_._5_-related secondary components, and atmospheric oxidation capacity were explored, and the causes of high NO_2_ values were investigated. The study results could provide a theoretical basis for NO_2_ pollution control and air quality improvement in Dongying and similar petrochemical cities.

## 2. Materials and Methods

### 2.1. Study Area and Data Sources

**Figure 1:**
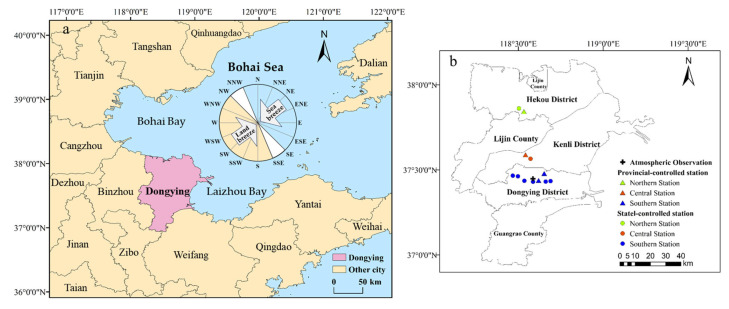
Geographical location of Dongying (**a**), and diagram of observation sites (**b**).

This study focuses on Dongying, a representative city in the Bohai Bay region, China. Dongying is located in the northwest of Shandong Province, bordered by Bohai to the east and north, adjacent to Binzhou City to the west, and neighboring Zibo and Weifang cities to the south (Figure 1a). As a typical resource-based city, Dongying is dominated by heavy industries (petroleum and chemicals), with industrial parks concentrated in the southern region. Consequently, the air quality in Dongying is easily influenced by both maritime and inland pollutant transports. Dongying has jurisdiction over five counties/districts: Dongying District, Hekou District, Kenli District, Lijin County, and Guangrao County. Currently, the city has one Super Atmospheric Observation Station (referred to as “Atmospheric Observatory”), eight state-controlled stations, and four provincial-controlled stations. The Hekou Urban Area and Hekou Development Zone stations are in the northern region, Minfeng Lake and Minfeng Road stations in Kenli District are in the central region, and the remaining stations are in the southern region (Figure 1b).

The routine observation items include NO_2_ and meteorological parameters—temperature (T), relative humidity (RH), WS, WD, atmospheric pressure (AP), and boundary layer heights (BLH). The enhanced observation items include routine monitoring parameters (NO_2_, NO, SO_2_, PM_2_._5_, O_3_), 115 VOCs, OC (organic carbon), EC (elemental carbon), ionic components (NO_3_^−^ and SO_4_^2−^), ultraviolet radiation (UR), and emissions data. All observation items were continuously monitored using automatic monitoring devices, with monitoring station, frequency and duration of data collection, quality assurance, and quality control adhering to various technical specifications. Details regarding data usage, sources, and quality control in this study can be found in Table S1, Text S1, and Text S2, respectively.

### 2.2. Related Definitions

NO_2_ evaluation standards: According to the Technical Regulation for Ambient Air Quality Assessment (on Trial) (HJ 663-2013), the daily NO_2_ assessment value is the average NO_2_ concentration over a 24 h period, the monthly NO_2_ assessment value is the average NO_2_ concentration for the month, and the annual NO_2_ assessment value is the average NO_2_ concentration for the calendar year. According to the Technical Regulation on Ambient Air Quality Index (on Trial) (HJ 633-2012), the daily NO_2_ concentration corresponding to the “Excellent” and “Good” air quality levels are 0–40 μg/m^3^ and 40–80 μg/m^3^, respectively. The daily assessment limit for NO_2_ under the National Ambient Air Quality Secondary Standard is 80 μg/m^3^. The World Health Organization (WHO) recommends annual NO_2_ Interim Target 2: IT-2 (30 μg/m^3^) and IT-3 (20 μg/m^3^). For 24 h NO_2_, IT-2 is 50 μg/m^3^, and the 2021 Air Quality Guideline (AQG) level is 25 μg/m^3^ [34].

The significance of NO_2_ at different percentiles: Analyzing the arithmetic mean of NO_2_ is complemented by evaluating NO_2_ at different percentiles to provide a more comprehensive and detailed description of NO_2_ pollution characteristics [35,36]. Generally, low percentiles of NO_2_ (5th percentile) represent background concentration, while high percentiles (99th and 95th percentiles) indicate concentration during severe NO_2_ pollution events [37]. Middle percentiles (75th, 50th, and 25th percentiles) reflect the NO_2_ concentration observed during most periods in Dongying.

High daily NO_2_ values (NO_2_-HD): NO_2_-HD is defined as daily average concentration exceeding the 75th percentile for each season in 2022, with seasonal thresholds of >23 μg/m^3^ (spring), >18 μg/m^3^ (summer), >45 μg/m^3^ (autumn), and >44 μg/m^3^ (winter) (Figure S2a). NO_2_-NHD is defined as the daily average concentration not exceeding the 75th percentile.

High hourly NO_2_ values (NO_2_-HH): NO_2_-HH is defined as hourly concentration exceeding the 75th percentile for each season in 2022, with seasonal thresholds of >25 μg/m^3^ (spring), >19 μg/m^3^ (summer), >47 μg/m^3^ (autumn), and >49 μg/m^3^ (winter) (Figure S2b).

NO_2_-HH events and NO_2_-NHH periods: Based on diurnal variation characteristics of NO_2_, NO_2_-HH typically occurs during nighttime to morning rush hours. NO_2_-HH events are defined as at least five consecutive NO_2_-HH occurrences during this period (with spring and summer from 00:00 to 09:00 and 20:00 to 23:00, and autumn and winter from 00:00 to 10:00 and 19:00 to 23:00) (Figure S8). Periods without five consecutive NO_2_-HH occurrences are defined as NO_2_-NHH periods. In addition, the remaining hours of each season are referred to as the daytime (with spring and summer from 10:00 to 19:00, and autumn and winter from 11:00 to 18:00).

### 2.3. Analytical Methods

#### 2.3.1. Methods for Analyzing Impact of NO_2_ on Air Quality

In this study, when analyzing the impact of NO_2_ on air pollution levels in Dongying, the number of mildly, moderately, heavily, and severely polluted days refers to the total number of polluted days where various basic pollutants were the primary pollutants, in accordance with the Technical Regulation on Ambient Air Quality Index (on Trial) (HJ 633-2012). The proportion of polluted days was then calculated as the ratio of the number of polluted days corresponding to a specific NO_2_ concentration range to the total number of all days in that range. To explore the impact of high NO_2_ values on different pollutants in detail, analyses were conducted from the perspectives of NO_2_-HH and NO_2_-HD in different seasons of Dongying. The analytical framework is detailed in Figure S3, and the main analytical methods are as follows:

Calculation of Secondary Organic Carbon (SOC): The concentration of SOC can be calculated using the OC/EC ratio method. The calculation formula is as follows:

SOC = OC − EC × (OC/EC)_min_ (1)

In the formula, SOC represents secondary organic carbon (µg/m^3^); OC and EC denote organic carbon and elemental carbon, respectively, and (OC/EC)_min_ represents the minimum value of the OC/EC ratio observed during the study period.

Evaluation indicators for atmospheric oxidation capacity: The atmospheric oxidation capacity is typically represented by O_X_ concentration, which is generally estimated using the concentration of NO_2_ and O_3_ (NO_2_ + O_3_). NO_3_ is an important nocturnal atmospheric oxidant, and the reaction between NO_3_ and NO_2_ is the only pathway for the formation of N_2_O_5_ [38]. The changes in N_2_O_5_ concentration levels can be indicated by the nighttime O_3_ concentration multiplied by the square of the NO_2_ concentration ([NO_2_]^2^ × O_3_), which indirectly represents the nighttime atmospheric oxidation capacity.

Transformation Rate of Nitrates and Sulfates: The nitrogen oxidation rate (NOR) and sulfur oxidation rate (SOR) can be used to assess the conversion status of gaseous precursors such as NO_2_ and SO_2_ into secondary inorganic ions. Higher values of NOR and SOR indicate a greater extent of secondary conversion of NO_2_ and SO_2_ in the atmosphere. The formulas are as follows:

NOR = N_1_/(N_1_ + N_2_) (2)

SOR = S_1_/(S_1_ + S_2_) (3)

In the formula, N_1_ and N_2_ represent the concentration of NO_3_^−^ and NO_2_, respectively (mol/m^3^); S_1_ and S_2_ represent the concentration of SO_4_^2−^ and SO_2_, respectively (mol/m^3^).

#### 2.3.2. Methods for Analyzing the Causes of High NO_2_ Values

The NO_2_ concentration distribution in 2022 is not significantly different from that in recent years. Additionally, the Atmospheric Observatory and meteorological station data for 2022 are relatively comprehensive. Therefore, this study selects 2022 for analyzing the causes of high NO_2_ levels. The analytical framework is detailed in Figure S4, and the main analytical methods are as follows:

Synoptic classification method: This study utilized the synoptic classification software cost733class developed by the European Union COST733 Project (http://www.cost733.org, accessed on 18 March 2024) and employed T-mode principal component Analysis (PCT) method to perform objective synoptic classification and reveal spatial distribution of the 925 hPa geopotential height field and the horizontal full WS (U and V) from 2017 to 2022. Details regarding the synoptic classification method in this study can be found in Text S3.

Criteria for sea–land breeze identification: Based on domestic and international studies on sea–land breeze classification standards [39,40], along with the natural geographic features of Dongying’s coastline, the criteria for identifying sea–land breeze events in Dongying are established as follows. Based on the orientation of the coastline, airflow within the SSE to NW range (157.5–315°) is defined as a land breeze, while airflow within the NNW to SE range (0–135° and 337.5–360°) is defined as a sea breeze (Figure 1a). A day is classified as a sea–land breeze day if the following criteria are met: (1) the 24 h average surface wind speed is below 10 m·s^−1^; (2) during the land breeze period (01:00–08:00), the land breeze duration is ≥4 h, while the sea breeze duration is ≤2 h; and (3) during the sea breeze period (13:00–20:00), the sea breeze duration is ≥4 h, while the land breeze duration is ≤2 h.

Random forest model: The random forest model, introduced by Breiman in 2001, is a machine learning algorithm based on classification trees. This model is widely used for handling nonlinear relationships, classification, regression, high-order correlations, and variable importance assessment [41,42]. A linear regression model and a random forest model were constructed using Python 3.13. The models randomly selected 90% of the data as the training set and the remaining 10% as the test set. Model performance was evaluated by calculating the coefficient of determination (R^2^) and root mean squared error (RMSE) [43]. Furthermore, the mean decrease accuracy (MDA) method was employed to evaluate the importance of each influencing factor. The principle behind MDA is that each variable is randomly assigned new values; if a variable is more important, replacing it randomly will result in a larger prediction error [44].

HYSPLIT mode: The Hybrid Single-Particle Lagrangian Integrated Trajectory Model (HYSPLIT) is primarily used to analyze air mass trajectories and trace the sources of air pollutants. In this study, the Dongying Atmospheric Observatory (37.45° N, 118.59° E) was selected as the starting point for the backward trajectories, with the analysis period covering the four seasons of 2022. The air mass analysis was conducted at an altitude of 300 m, with a backward tracing duration of 24 h. A total of 24 trajectories were calculated per day, resulting in 2208, 2206, 2184, and 2160 effective trajectories for each season. More details can be found in Text S4.

Backward trajectory clustering analysis method: Backward trajectory clustering analysis uses mathematical methods to classify and cluster all air parcel trajectories within a set time based on transport speed and direction. This helps analyze the source and proportion of dominant air masses at the target location, thereby determining the primary pollution source direction [45]. After cluster analysis, this study statistically analyzed the trajectory types and corresponding NO_2_ hourly concentration data for each season, and subsequently determined the mean NO_2_ concentration and contribution percentage for each trajectory type. More details can be found in Text S5. The formula for calculating the contribution percentage of NO_2_ concentration is as follows.

(4) $$P_{{NO}_{2},t}=\frac{\sum C_{t}}{\sum C}\times 100\%$$

t is the category corresponding to the trajectory, $C_{t}$ is the hourly mass concentration of NO_2_ corresponding to the trajectory with category t, $\sum C_{t}$ is the sum of hourly mass concentrations of NO_2_ corresponding to the trajectory with category t, ∑C is the sum of hourly mass concentrations of NO_2_ of all the trajectories, and $P_{{NO}_{2},t}$ is the contribution of NO_2_ concentration corresponding to the category of trajectory, t.

Potential source contribution function (PSCF) analysis method: PSCF is a method based on conditional probability functions to identify potential pollution sources [46]. This method relies on backward trajectory results and utilizes a series of functions in the TrajStat plugin to calculate WPSCF values. In this study, NO_2_-HH in each season were set as the threshold for the target pollutant. The higher the WPSCF value in an area, the darker the color in the generated graphics, indicating a higher probability that the area contributes to NO_2_-HH at the target site. More details can be found in Text S6.

Concentration weight trajectory (CWT) analysis method: The CWT method calculates the weighted concentration of trajectories to quantitatively reflect the pollution severity of different trajectories [47], thus determining the impact of regional transport on NO_2_ concentration in Dongying. The higher the WCWT value, the more pollutants transported by the trajectory passing through the grid to the target site, and the greater the contribution to NO_2_ concentration at the target site [48]. More details can be found in Text S7.

## 3. Results and Discussion

### 3.1. NO_2_ Concentration Levels and Variation Characteristics

#### 3.1.1. Concentration Levels

**Figure 2:**
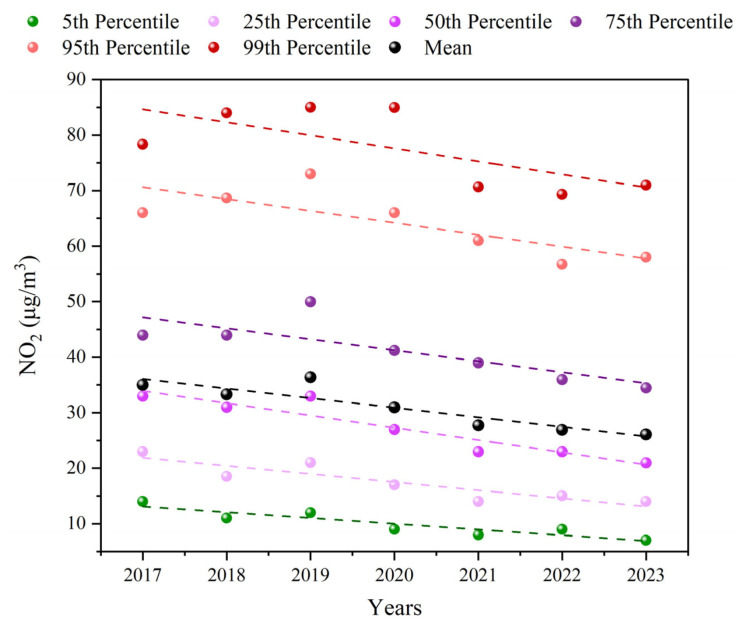
Variations in different percentiles and the annual mean values of the daily average NO_2_ concentrations in Dongying from 2017 to 2023.

From 2017 to 2023, the annual mean values of NO_2_ in Dongying showed a trend of “increase-decrease-stabilization” (Figure 2). The 5th, 25th, 50th, and 75th percentiles of NO_2_ in Dongying exhibited a clear downward trend overall, except for a rise in 2019. The 95th and 99th percentiles of NO_2_ displayed a fluctuating trend of “increase–decrease–increase,” indicating a significant difference in the trends between low and high percentiles of NO_2_. Additionally, in 2023, while the low and middle percentiles continued to decline and the high percentiles rebounded, the annual average remained relatively stable. This indicates that the high NO_2_ concentration issue in Dongying has not been resolved. The NO_2_ concentration at the 95th percentile in Dongying each year was consistently higher than that at the 75th percentile, further indicating a high NO_2_ concentration issue. Notably, in 2019, the annual mean values of NO_2_ were the highest, and both the 75th and 95th percentiles were significantly elevated compared to other years. This suggests that the high NO_2_ concentration in 2019 may have been driven by increases in the middle and high percentiles. In recent years, the annual NO_2_ concentration in Dongying has met IT-2 (30 μg/m^3^), but there is still a significant gap in reaching IT-3 (20 μg/m^3^). In summary, Dongying currently faces a significant issue with high NO_2_ concentration, and reducing the high values could notably lower the overall NO_2_ concentration levels in the city.

#### 3.1.2. Temporal Variation Characteristics

**Figure 3:**
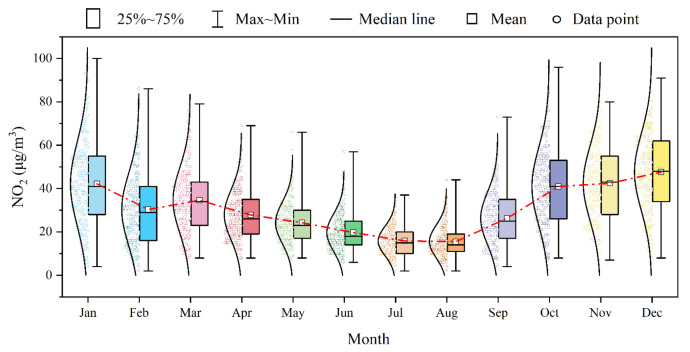
Monthly variation in the daily average NO2 concentrations in Dongying from 2017 to 2023.

**Figure 4:**
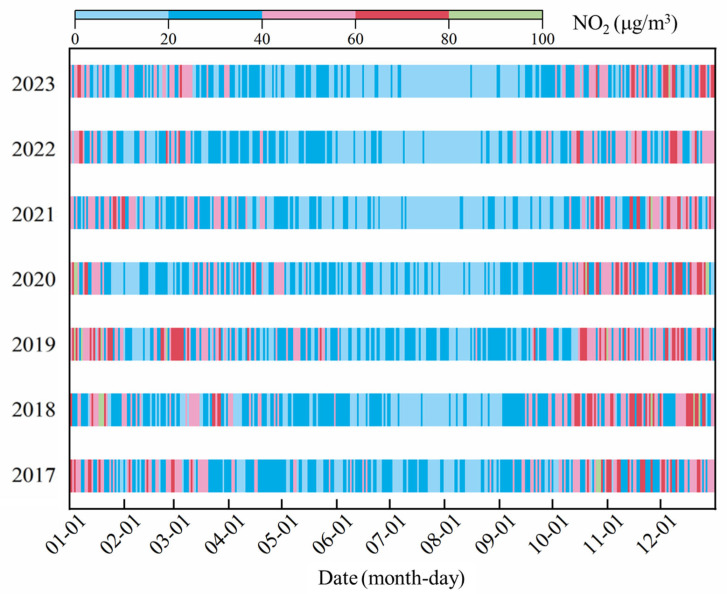
NO_2_ concentrations calendar in Dongying from 2017 to 2023.

The monthly variation of NO_2_ in Dongying exhibits a “U” distribution (Figure 3), which is consistent with the findings of most researchers [49,50]. Overall, NO_2_ concentration was highest in December, followed by November, October, and January. A significant decline in NO_2_ concentration was observed each year from January to February. The period from March to August marked a rapid decrease in NO_2_ levels, reaching the lowest values in July and August, while the period from August to October shows a rapid increase. The calendar of NO_2_ in Dongying from 2017 to 2023 (Figure 4) shows that NO_2_ with high concentrations (>40 μg/m^3^) was concentrated from January to mid-March and from September to late December in 2017, with exceedances (>80 μg/m^3^) occurring in late October. Compared to 2017, NO_2_ with high concentrations in 2018 was concentrated from October to December and March. The days in January and February were more dispersed, but days where exceedances occurred in mid-January and were concentrated. In 2019, compared to 2018, the days of NO_2_ with high concentrations increased and became more concentrated, with exceedance days mainly occurring in January and being more dispersed. From 2020 to 2023, the days of NO_2_ with high concentrations significantly decreased and became more dispersed. In 2023, Dongying still experienced 46 days with NO_2_ concentrations exceeding IT-2 (50 μg/m^3^) and 159 days not meeting the 2021 AQG level (25 μg/m^3^), indicating a relatively high daily average NO_2_ level in the city. Overall, the days of NO_2_ with high concentrations in Dongying exhibited a distribution pattern of “few and dispersed days—increasing and concentrated days—gradually decreasing and more dispersed days” from 2017 to 2023. High NO_2_ concentrations mainly occurred from September to December and January to April, with exceedances usually happening in late October, November, December, and January.

**Figure 5:**
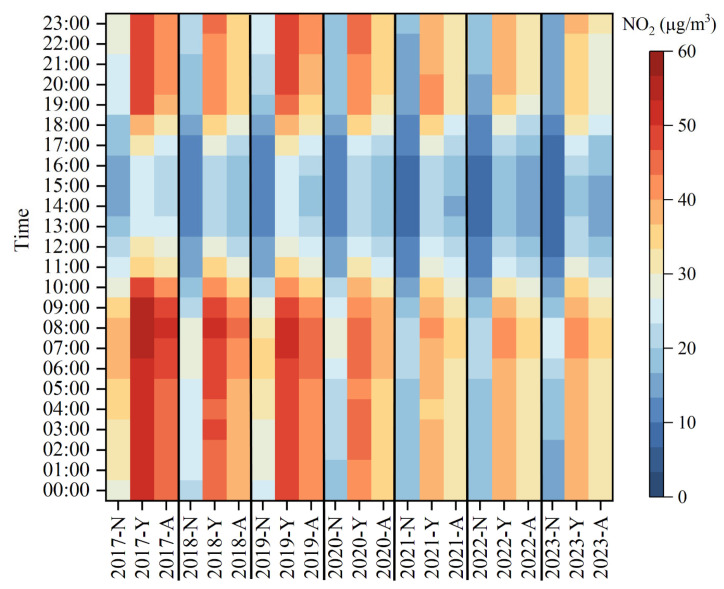
Diurnal variation of NO_2_ concentrations in Dongying from 2017 to 2023. (“Year-N” represents the NO_2_ concentration during non-high NO_2_ concentrations periods of a specific year, “Year-Y” represents the NO_2_ concentration during high NO_2_ concentrations periods of a specific year, and “Year-A” represents the annual mean NO_2_ concentration of a specific year).

From 2017 to 2023, the concentrations of NO_2_ during high NO_2_ concentrations periods (from September to December and January to April) and non-high NO_2_ concentrations periods, and the annual mean value of NO_2_ concentration in Dongying all exhibited a “single peak and single valley” pattern (Figure 5 and Figure S5). The peak occurred around 8:00 a.m., and the valley appeared around 2:00 PM. During the morning traffic rush (6–9 a.m.), NO_2_ concentration slightly increased, then slowly decreased after 9 a.m. After 4:00 p.m., NO_2_ concentration rose significantly, remained at high levels, and exhibited a slow upward trend during nighttime to morning rush hours. Most research findings indicate that daily NO_2_ variations exhibit a “double peak and single valley” pattern, with distinct morning and evening peaks [23,51]. In comparison, the hourly NO_2_ concentration in Dongying did not show a significant peak at night, and the morning peak was smaller, indicating that in addition to vehicle emissions, other pollution sources also influence NO_2_ concentration in Dongying. Overall, from 2017 to 2023, the concentrations of NO_2_ during high NO_2_ concentrations periods and non-high NO_2_ concentrations periods, and the annual mean value of NO_2_ concentration all showed a downward trend. However, changes in NO_2_ concentration mainly occurred during nighttime to morning rush hours, with minimal changes during daytime hours.

#### 3.1.3. Spatial Variation Characteristics

**Figure 6:**
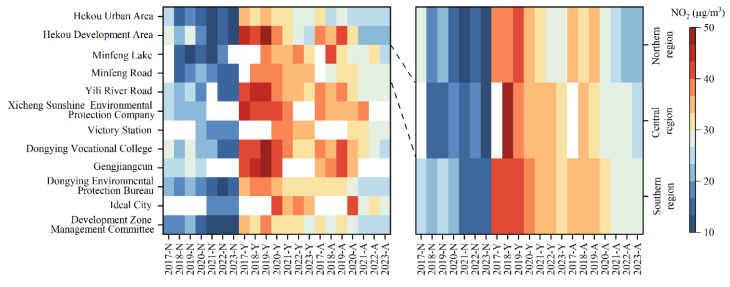
Interannual variation of NO_2_ concentrations in different regions of Dongying from 2017 to 2023. (“Year-N” represents the NO_2_ concentration during non-high NO_2_ concentrations periods of a specific year, “Year-Y” represents the NO_2_ concentration during high NO_2_ concentrations periods of a specific year, and “Year-A” represents the annual mean NO_2_ concentration of a specific year. Blank sections indicate non-operational monitoring sites or missing data).

The interannual variation of NO_2_ concentration in different regions of Dongying from 2017 to 2023 is shown in Figure 6. From different monitoring sites, during non-high NO_2_ concentrations periods, the highest average NO_2_ concentration was at the Gengjiangcun site, and the lowest was at the Minfeng Lake site in Kenli District. During periods of high NO_2_ concentrations, the highest average NO_2_ concentration was also at the Gengjiangcun site, while the lowest was at the Hekou urban site. From the annual variation at different sites, the Gengjiangcun site consistently showed higher average NO_2_ concentration, while the Hekou urban site had lower averages. From different regions, during non-high NO_2_ concentrations periods, the southern region had the highest average NO_2_ concentration (19 μg/m^3^), and the central region had the lowest (15 μg/m^3^). During high NO_2_ concentrations periods, the southern region had the highest average NO_2_ concentration (37 μg/m^3^), while the northern region had the lowest (34 μg/m^3^). From the annual variation at different regions, the southern region had the highest average NO_2_ concentration (19 μg/m^3^), while the central region had the lowest (15 μg/m^3^). Overall, from the perspectives mentioned above, the NO_2_ concentration levels in the southern region of Dongying are relatively higher compared to other regions.

### 3.2. Impact of NO_2_ on Air Quality

#### 3.2.1. Impact of NO_2_ on Air Quality Levels

From the perspective of the primary pollutant (Figure S6), the number of days with NO_2_ as the primary pollutant in Dongying was the lowest in 2017 (1 d), higher from 2018 to 2020 (14–20 days), and relatively lower from 2021 to 2023 (10–13 days) with a fluctuating trend. The proportion of days with NO_2_ daily assessment value of “excellent” showed an overall increasing trend, reaching its lowest point in 2019 (64%) and experiencing a slight rebound in 2023. Exceedance days for NO_2_ occurred between 2017 and 2020 (3–5 days) and in 2023 (1 day). Overall, as a pollutant in air quality assessment, NO_2_ itself currently has a reduced impact on the air quality in Dongying.

**Figure 7:**
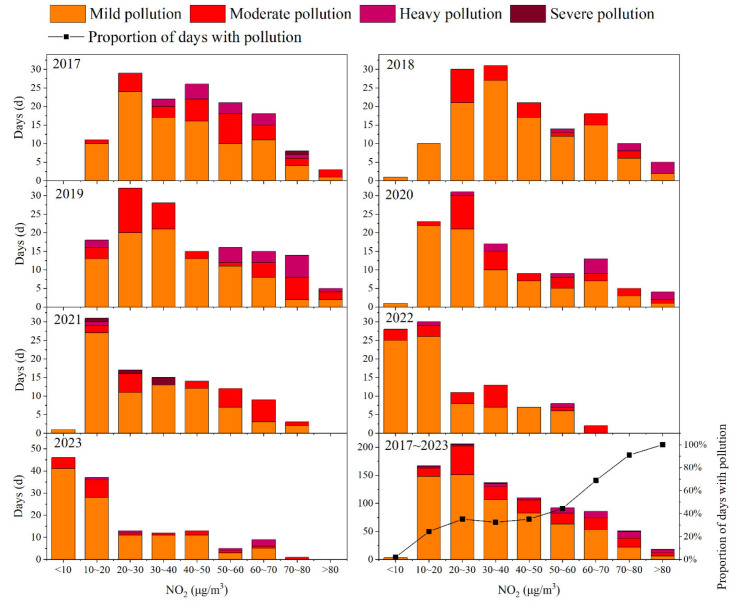
Distribution of air pollution levels corresponding to NO_2_ daily average concentration ranges in Dongying from 2017 to 2023.

According to Figure 7, from 2017 to 2019, the number of mildly polluted days generally increased and then gradually decreased with rising NO_2_ concentration. From 2020 to 2023, the number of mildly polluted days steadily decreased with rising NO_2_ concentration. The number of moderately polluted days showed an increase, followed by a decrease and then another increase with rising NO_2_ concentration. Severe pollution days mainly occurred in 2017 (1 day) and 2021 (4 days), with corresponding NO_2_ concentration ranges of 70–80 and 30–40 µg/m^3^, respectively. Unlike other polluted days, the number of severe pollution days is relatively small, and there is no obvious pattern of variation. From 2017 to 2023, the number of mildly, moderately, and heavily polluted days was higher when the NO_2_ concentration were 10–30 μg/m^3^, 20–30 μg/m^3^, and 50–80 μg/m^3^, respectively. When the NO_2_ concentration is below 20 μg/m^3^, the proportion of polluted days is relatively low (<25%). Within the 20–50 μg/m^3^ range, the proportion remained stable (32–35%). However, when the NO_2_ concentration exceeded 60 μg/m^3^, the proportion of polluted days increased significantly (>60%). In summary, the overall air quality in Dongying showed that both the air pollution level and the proportion of polluted days increased with rising daily NO_2_ concentrations.

#### 3.2.2. Impact of High NO_2_ Values on O_3_, PM_2_._5_, and Atmospheric Oxidation Capacity in 2022

According to Figure S7, the number of NO_2_-HD days in Dongying in 2022 showed no significant seasonal variation, with similar counts in spring (21 days), summer (21 days), autumn (19 days), and winter (22 days). On a monthly scale, December and March had the highest number of NO_2_-HD days (11 days), followed by June and November (9 days), while February, April, and September had the fewest (4 days), indicating some inter-monthly variability. As shown in Figure S8, the proportion of NO_2_-HH in spring and summer peaked around 00:00–01:00 (8% and 7%) and again around 07:00 (8% and 10%). In contrast, NO_2_-HH in autumn and winter remained relatively stable between 19:00 and 10:00, the following day, ranging from 5% to 7% and 4% to 6%, respectively. Overall, NO_2_-HH in Dongying primarily occurred from nighttime to the morning peak hours (with spring and summer being 00:00–09:00 and 20:00–23:00, and autumn and winter 00:00–10:00 and 19:00–23:00).

**Figure 8:**
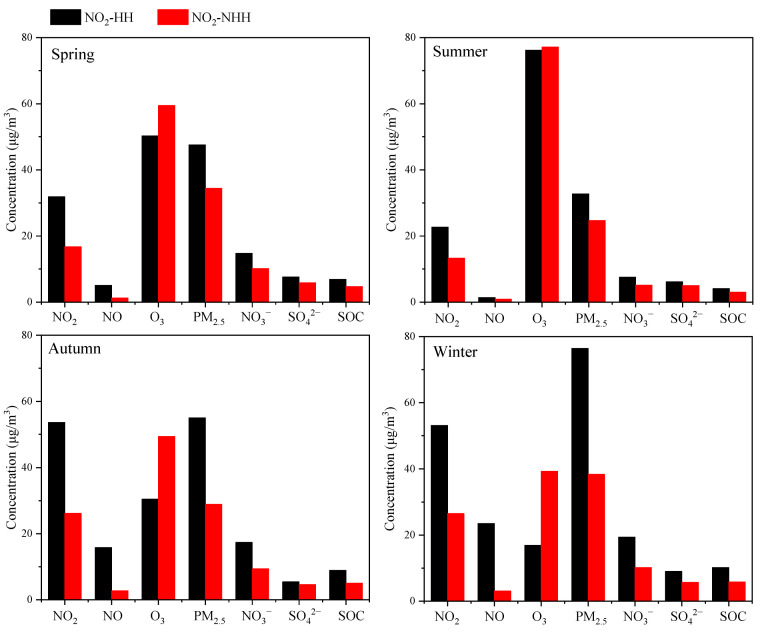
Differences in the concentrations of O_3_, PM_2.5_ and PM_2.5_-bounded secondary components during nighttime to morning rush hours between NO_2_-HH events and NO_2_-NHH periods in Dongying in 2022.

**Figure 9:**
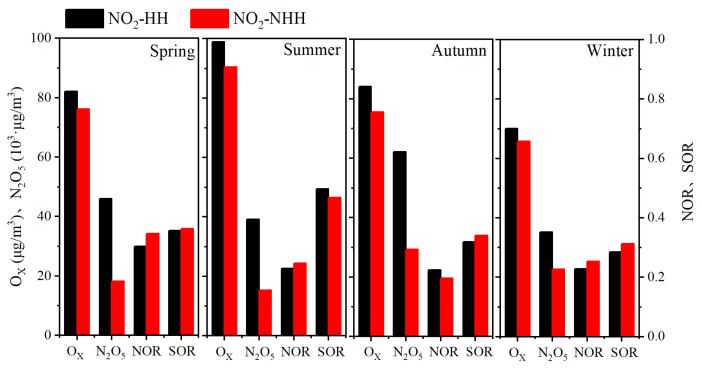
Differences in the concentrations of O_X_ and N_2_O_5_, and values of NOR and SOR during nighttime to morning rush hours between NO_2_-HH events and NO_2_-NHH periods in Dongying in 2022.

As shown in Figure 8, compared to NO_2_-NHH periods, NO_2_-HH events in all seasons lead to significant increases in PM_2_._5_ and PM_2.5_-bounded NO_3_^−^, SO_4_^2^^−^, and SOC concentrations, and a notable decrease in O_3_ concentration. This indicates that high NO_2_ values can enhance the concentrations of O_3_, PM_2.5_ and PM_2.5_-bounded secondary components during nighttime to morning rush hours, with the most pronounced effects in autumn and winter. The higher the NO_2_ concentration in a season, the lower the nighttime O_3_ concentration, which is related to the NOx titration reaction [2]. As shown in Figure 9, during NO_2_-HH events across all seasons, the concentrations of O_X_ and N_2_O_5_ are significantly higher compared to NO_2_-NHH periods, indicating that high NO_2_ values can enhance the atmospheric oxidation capacity at night, consistent with the findings of Alberto Notario et al. [52]. In spring, summer, and winter, the NOR values decrease during NO_2_-HH events, whereas the opposite is observed in autumn. Similarly, SOR values decrease during NO_2_-HH events in spring, autumn, and winter, but increase in summer. This may be because the formation of NO_3_^−^ and SO_4_^2^^−^ at night is significantly influenced by nighttime O_3_ concentration [53]. During NO_2_-HH events, high NO concentration results in the titration of O_3_ by NO and the concentrations of NO_2_ and SO_2_ are relatively high, leading to lower NOR and SOR values. In summary, NO_2_-HH events can increase O_3_, PM_2.5_ and PM_2.5_-bounded secondary components concentrations, as well as O_X_ and N_2_O_5_ concentrations during nighttime to morning rush hours. They also lead to lower O_3_ concentration, NOR, and SOR values. Moreover, the analysis of impact of high NO_2_ values on daytime O_3_, PM_2.5_, and atmospheric oxidation capacity in Dongying in 2022 is detailed in Figures S9 and S10 and Text S8.

**Figure 10:**
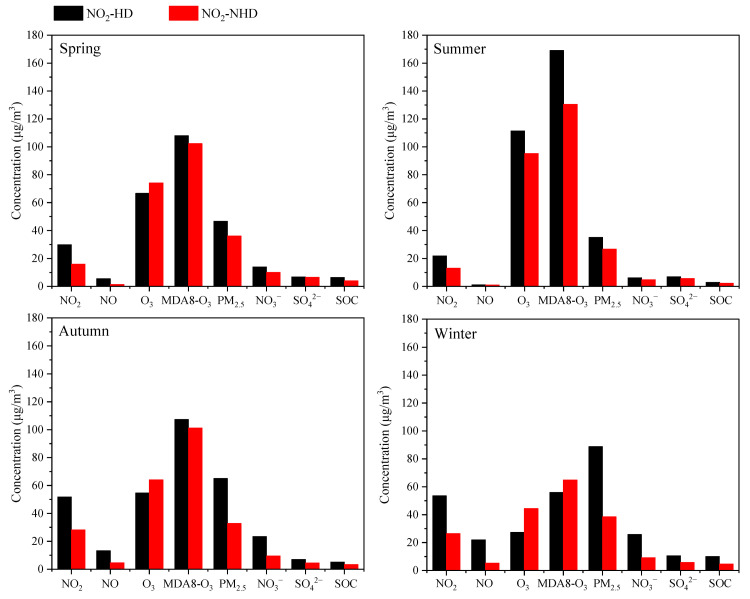
Differences in the concentrations of O_3_, PM_2.5_ and PM_2.5_-bounded secondary components on the same day between NO_2_-HD days and NO_2_-NHD days in Dongying in 2022.

**Figure 11:**
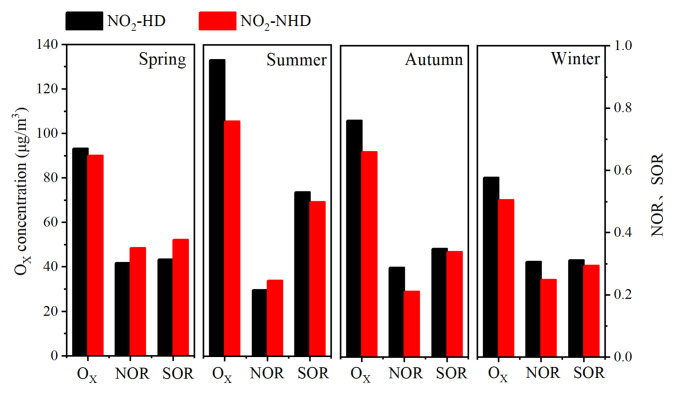
Differences in the concentrations of O_X_, and values of NOR and SOR on the same day between NO_2_-HD days and NO_2_-NHD days in Dongying in 2022.

As shown in Figure 10, compared to NO_2_-NHD days, PM_2.5_ and PM_2.5_-bounded NO_3_^−^, SO_4_^2^^−^, and SOC concentrations are significantly higher on NO_2_-HD days across all seasons, indicating that NO_2_-HD can promote increases in concentrations of PM_2.5_ and PM_2.5_-bounded secondary components on the same day, with the most pronounced effect in autumn and winter. In spring, autumn, and winter, O_3_ concentration on NO_2_-HD days is lower than on NO_2_-NHD days, whereas the opposite is true in summer. In spring, summer, and autumn, MDA8-O_3_ concentration on NO_2_-HD days is higher than on NO_2_-NHD days, with the opposite observed in winter, likely due to higher daytime NO emissions in winter. As shown in Figure 11, O_X_ concentration is significantly higher on NO_2_-HD days across all seasons, indicating that NO_2_-HD can enhance the atmospheric oxidation capacity. In spring and summer, NOR values on NO_2_-HD days are lower than on NO_2_-NHD days, whereas the opposite is observed in autumn and winter. Conversely, SOR values are higher on NO_2_-HD days in summer, autumn, and winter, but lower in spring, indicating a noticeable seasonal difference in the impact of NO_2_-HD on NOR and SOR. In summary, NO_2_-HD can increase MDA8-O_3_, PM_2.5_ and PM_2.5_-bounded secondary component concentrations, as well as O_X_ concentration, while reducing the average O_3_ concentration on the same day.

### 3.3. Causes of High NO_2_ Values

#### 3.3.1. Meteorological Conditions

Using the PCT method, the circulations of the 925-hPa geopotential height field from 2017 to 2022 were classified into nine patterns (Figure S11)—namely, “offshore high-pressure rear” (Type 1), “high-pressure front” (Type 2), “high-pressure inside” (Type 3), “high-pressure top front” (Type 4), “uniform pressure” (Type 5), “west-high and east-low” (Type 6), “subtropical high” (Type 7), “inverted trough” (Type 8), and “low-pressure field” (Type 9).

From 2017 to 2022, Type 1 occurs most frequently (20.5%), with large variations in the daily average maximum temperature (T_max_-mean) and daily average relative humidity (RH-mean). Type 4 (16.6%), Type 2 (14.1%), and Type 3 (12.1%) followed, with these patterns generally characterized by low T_max_-mean and high RH-mean. Type 6 (8.4%) and Type 5 (6.7%) exhibited large ranges in T_max_-mean, with the former having sparse isobars and lower RH-mean, and the latter having dense isobars and higher RH-mean. Type 7 (9.5%), Type 8 (7.6%), and Type 9 (4.4%) mainly occur in summer, characterized by higher T_max_-mean, occasional precipitation, and higher RH-mean (Table 1).

**Figure 12:**
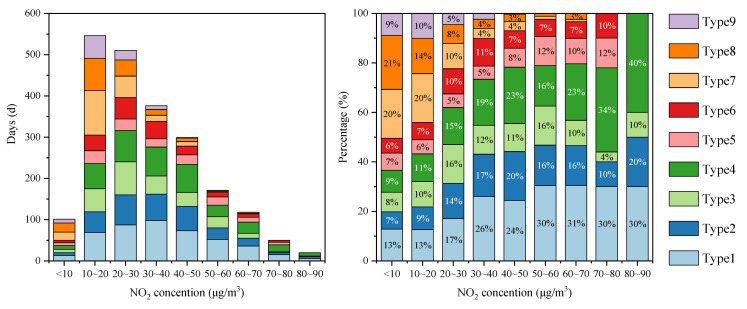
Frequencies and percentages of different synoptic patterns corresponding to NO_2_ daily average concentration ranges in Dongying from 2017 to 2022.

As shown in Figure 12, when the proportion of “subtropical high” (Type 7), “inverted trough” (Type 8), and “low-pressure field” (Type 9) was higher, the NO_2_ concentration in Dongying was relatively low. Conversely, when the proportion of these types decreased and the proportion of high-pressure synoptic patterns (Type 1, Type 2, Type 3, and Type 4) increased, the NO_2_ concentration rose. This is because Type 7, Type 8, and Type 9 usually occur in summer, with higher T_max_-mean, occasional precipitation, and higher RH-mean, which facilitate the removal of pollutants. When Dongying was under “offshore high-pressure rear” (Type 1), the impact of the cold high-pressure was waning, resulting in stable synoptic conditions and low RH, which can lead to a higher concentration of accumulated pollutants and higher NO_2_ levels. When Dongying was under “high-pressure front” (Type 2) and “high-pressure top front” (Type 4), the passage of cold air masses resulted in a significant drop in T and RH. Additionally, the dominant wind was typically a low-speed southwest wind, which was conducive to the increase in NO_2_ concentration. When Dongying was under “high-pressure inside” (Type 3), the activity of the cold high-pressure was weak, leading to conditions such as temperature inversion, which can result in NO_2_ exceedances. Moreover, when Dongying was under “uniform pressure” (Type 5), horizontal atmospheric movement is weak, often accompanied by strong radiative inversions or low-level inversions, making high NO_2_ concentration more likely [54]. The above analysis indicates that Type 7, Type 8, and Type 9 help to reduce NO_2_ concentration, whereas high-pressure synoptic patterns (Type 1, Type 2, Type 3, and Type 4) and “uniform pressure” (Type 5) tend to trigger NO_2_ pollution.

**Figure 13:**
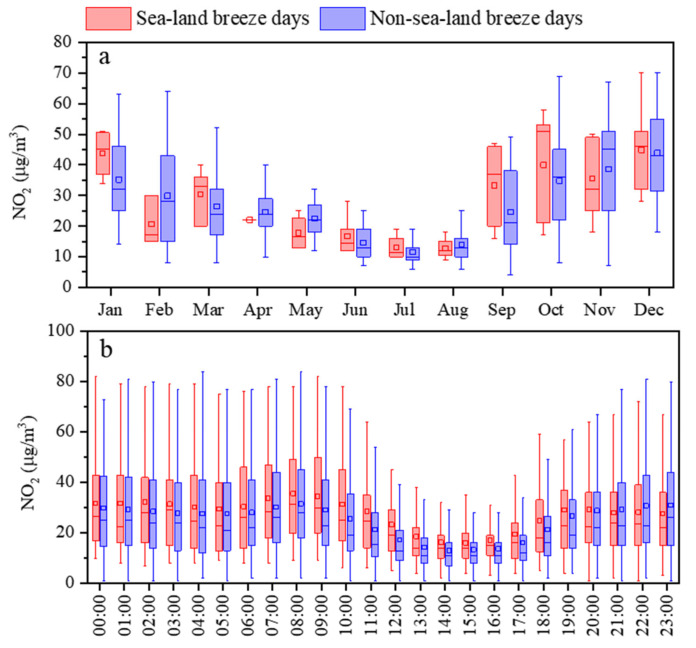
The monthly (**a**) and daily (**b**) variation in NO_2_ concentrations on land–sea breeze and non-land–sea breeze days in Dongying in 2022.

The monthly variation in NO_2_ concentrations on land–sea breeze and non-land–sea breeze days in Dongying City in 2022 is shown in Figure 13a. Overall, the average NO_2_ concentration on land–sea breeze days in Dongying (29.1 µg/m^3^) was higher than that on non-land–sea breeze days (26.5 µg/m^3^). In January, March, and September to October, the NO_2_ concentration on land–sea breeze days in Dongying was higher than that on non-land–sea breeze days. From June to August and November to December, the NO_2_ concentrations on land–sea breeze days were similar to those on non-land–sea breeze days. In February and from April to May, the NO_2_ concentration on land–sea breeze days was lower than that on non-land–sea breeze days. The above analysis indicates that during high NO_2_ concentrations periods, the land–sea breeze has a significant contribution to the elevated daily average NO_2_ concentrations in Dongying City.

The daily variation in NO_2_ concentrations on land–sea breeze and non-land–sea breeze days in Dongying City in 2022 is shown in Figure 13b. Overall, the hourly NO_2_ concentrations on land–sea breeze days were significantly higher than those on non-land–sea breeze days. From 00:00 to 20:00, the NO_2_ concentrations on land–sea breeze days were higher than those on non-land–sea breeze days. From 00:00 to 04:00, the difference between the two was small, ranging from 1.9 to 3.6 µg/m^3^. From 05:00 to 15:00, the difference gradually increased and then decreased, with the greatest difference observed at 11:00 (7.3 µg/m^3^). From 16:00 to 18:00, the concentration difference between the two remained between 3.3 and 3.5 µg/m^3^. From 19:00 to 20:00, the difference gradually decreased, and by 21:00, the NO_2_ concentration on land–sea breeze days was lower than on non-land–sea breeze days. The possible explanation for this phenomenon is as follows: during the early phase of land breeze dominance, pollutants from the continent are transported to the air over the Bohai Sea. Meanwhile, ship emissions in the Bohai region also contribute to air pollution [55], and currently, NOx and VOC emissions from ships in China remain relatively high [56]. Consequently, NO_2_ gradually accumulates over the Bohai Sea. During the transition from land breeze to sea breeze, NO_2_ pollution plume over the Bohai Sea are gradually transported back to Dongying City. Under the combined influence of transport and local emissions, the hourly NO_2_ concentration is higher than that on non-land–sea breeze days [20]. During the period of sea breeze dominance, NO_2_ is continuously transported from the Bohai Sea to Dongying, resulting in a large difference between the two. During the transition from sea breeze to land breeze, the NO_2_ concentration in the sea area decreases and is gradually carried back to the air over the Bohai Sea by the dominant land breeze, resulting in a smaller difference in NO_2_ concentrations between land–sea breeze and non-land–sea breeze days, with the concentration on land–sea breeze days at certain hours being lower.

**Figure 14:**
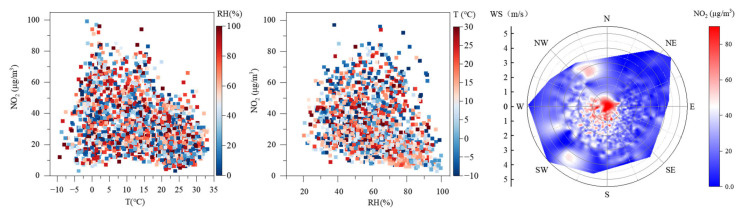
Variation of NO_2_ concentrations with T, RH, WS, and WD in Dongying from 2017 to 2022.

The variation of NO_2_ concentrations with T, RH, WS, and WD in Dongying from 2017 to 2022 is shown in Figure 14. High NO_2_ concentration (>40 μg/m^3^) are notably concentrated when T from −5 to 15 °C and RH from 30 to 80%. Daily average NO_2_ concentration exceeding standards is more likely when T are below 10 °C and RH ranges from 50 to 70%. It is noteworthy that NO_2_ concentration does not show a strong linear relationship with T and RH. This can be explained by the findings of Huang et al. [57], who, through extensive data analysis, discovered that NO_2_ concentrations are primarily influenced by BLH and AP, especially by BLH. When WS in Dongying are below 1 m/s, both the level of daily average NO_2_ concentration and the frequency of high NO_2_ concentration are relatively high. When WD is N to NW and WS range from 2 to 3 m/s, high NO_2_ concentration are more frequent. When WD is W to S, the level of daily average NO_2_ concentration and the frequency of high NO_2_ concentration decrease with increasing WS. Overall, high NO_2_ concentration are more likely to occur when WS are below 1 m/s. While when WS exceeds 1 m/s, southwestern winds facilitate a higher NO_2_ concentration. This may be due to local pollutant accumulation at low WS and regional pollutant transport at high WS [58,59].

In addition, this study analyzed the correlation between the daily average NO_2_ concentrations and meteorological factors from 2017 to 2022 (Table S2). From a seasonal perspective, WS (−0.18 to −0.57) and RH (−0.30 to −0.60, except in winter) exhibited relatively consistent correlations across all seasons, whereas T and AP showed seasonal variations. Previous studies have indicated that the strength and correlation of meteorological factors affecting NO_2_ concentrations vary depending on the season [60] or the study period [61]. From an annual perspective, NO_2_ concentrations showed a significant negative correlation with WS (−0.43), T (−0.45), and RH (−0.38), while exhibiting a positive correlation with AP (0.46). These results are consistent with findings from Handan [62], as well as studies in Shijiazhuang, Foshan and Ya’an regarding WS, T and AP, although no significant correlation with RH was observed in these regions [60,63,64]. The possible reasons for these findings include higher NO_2_ emissions in winter, while elevated T in summer enhances photochemical consumption; low WS hinders pollutant dispersion; high RH may promote the heterogeneous reaction of NO_2_ [65]; and high AP leads to descending airflows and suppresses pollutant dispersion.

In summary, high NO_2_ concentrations in Dongying tend to occur under meteorological conditions characterized by relatively low T (−5 to 15 °C), moderate RH (30–80%), southwesterly WD, and WS below 1 m/s.

**Figure 15:**
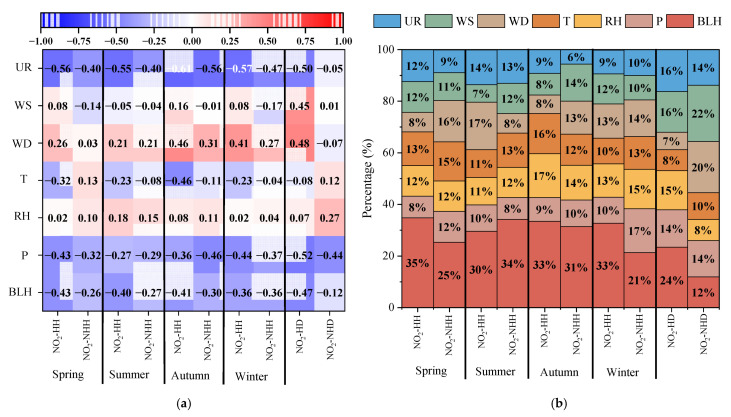
The correlation between NO_2_ and meteorological factors (**a**), and the importance share of each factor (**b**) on days with NO_2_-HH events and NO_2_-NHH periods in each season of Dongying in 2022.

The factor importance obtained after building a random forest model based on meteorological data is shown in Figure 15. Overall, the main factors influencing the hourly NO_2_ concentrations in Dongying City in 2022 were BLH, while the dominant factors for the daily average NO_2_ concentrations were BLH, WS, and UR. Studies have shown that a decrease in BLH is a key reason for the significant increase in NO_2_ concentrations at night and across seasons [66,67]. From the difference between NO_2_-HH and NO_2_-NHH, the importance of BLH significantly increased during the spring and winter for NO_2_-HH (with correlations of −0.43 and −0.36, and importance contribution differences of 10% and 12%, respectively). This is because BLH is generally lower in spring and winter, which facilitates the occurrence of high NO_2_ values, thus contributing to the main cause of NO_2_-HH formation. In summer, the importance of WD increased significantly (correlation of 0.21, importance contribution difference of 10%), likely due to the transport of NO_2_ air masses in Dongying City, which is a major factor in the formation of NO_2_-HH. In autumn, the importance of T increased significantly (correlation of −0.46, importance contribution difference of 4%), which is related to Dongying City’s low-temperature environment, making it conducive to the formation of NO_2_-HH. From the difference between NO_2_-HD and NO_2_-NHD, the importance of BLH significantly increased, indicating that BLH is the primary factor for the occurrence of NO_2_-HD.

#### 3.3.2. Chemical Generation

**Figure 16:**
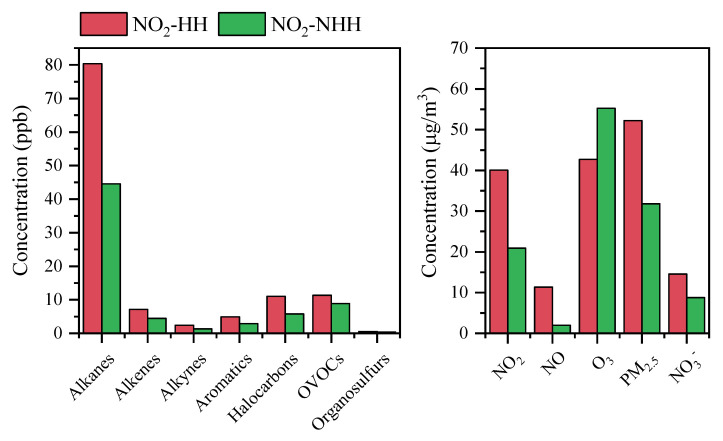
Differences in concentrations of different types of VOCs and related pollutants during nighttime to morning rush hours between NO_2_-HH events and NO_2_-NHH periods in Dongying in 2022.

The chemical reactions between NO, NO_2_, VOCs, and O_3_ affect the concentration of NO_2_. As shown in Figure 16, compared to NO_2_-NHH periods, all types of VOCs increase during NO_2_-HH events, with alkanes and halogenated hydrocarbons showing a more pronounced increase, while OVOCs increase to a lesser extent. This suggests that the significant increase in VOCs during NO_2_-HH events, particularly alkynes, alkanes, and alkenes, may be associated with the formation of NO_2_-HH. In terms of changes in pollutant concentrations, except for a marked decrease in O_3_ concentration during NO_2_-HH events, concentrations of other pollutants are higher, with NO showing the greatest increase. This is related to NO_2_-related chemical reactions, particularly the titration reaction between NO and O_3_ and the series of nighttime chemical reactions involving NO_2_ [3]. The balance of these reactions significantly influences hourly NO_2_ concentration levels. Overall, NO_2_-HH events are more likely to occur when VOCs and NO concentrations are high, and O_3_ concentration is low.

**Figure 17:**
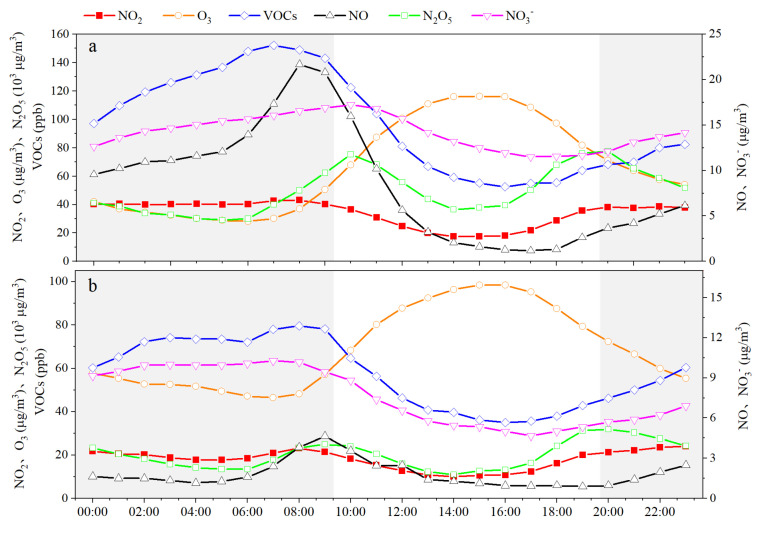
Diurnal variations in different air pollutants on days with NO_2_-HH events (**a**) and NO_2_-NHH periods (**b**) in Dongying in 2022.

As shown in Figure 17, on NO_2_-HH event days, VOCs and NO concentrations increase significantly during nighttime and morning rush hours, exhibiting a unimodal pattern, with more pronounced photochemical consumption at noon. In contrast, on NO_2_-NHH days, VOCs and NO concentrations remain relatively stable at night, with a smaller increase during the early morning rush and less photochemical consumption at noon. The high concentrations of VOCs and NO in Dongying contribute to intense photochemical reactions during the day, leading to higher NO_2_ levels. On NO_2_-HH event days, the peak NO concentration in the evening is higher, which rapidly reduces nighttime O_3_ levels through titration reactions, while the NO_2_ concentration remains stable after a rapid increase. Xia et al. [2] found that an increase in the diurnal variation of NO_2_ is accompanied by a larger diurnal variation in O_3_, which is consistent with the variations in NO_2_ and O_3_ on NO_2_-HH event days in Dongying. Additionally, studies have shown that when NO concentration is high, NO_3_ is primarily removed through reaction with NO to form NO_2_, which suppresses the nighttime NO_2_ net consumption rate about NO_3_. Therefore, the NO_3_^-^ formation rate is lower during the evening peak of NO_2_-HH event days. The stability of NO_2_ in the early morning indicates that on NO_2_-HH event days, there is ample NO, and NO_2_ is in a dynamic equilibrium between generation and consumption, with O_3_ continuously depleted and NO_3_^−^ increasing. Conversely, on NO_2_-NHH days, after the NO_2_ concentration rises during the evening peak, it shows a decreasing trend along with VOCs in the early morning. This is due to insufficient NO at night and higher O_3_ concentration, which dominate the NO_2_ net consumption reactions. Overall, daytime NO_2_ concentration are primarily influenced by intense photochemical reactions, with higher VOCs and NO concentrations leading to higher daytime NO_2_ levels. During nighttime and morning rush hours, NO and O_3_ significantly affect NO_2_ concentration.

**Figure 18:**
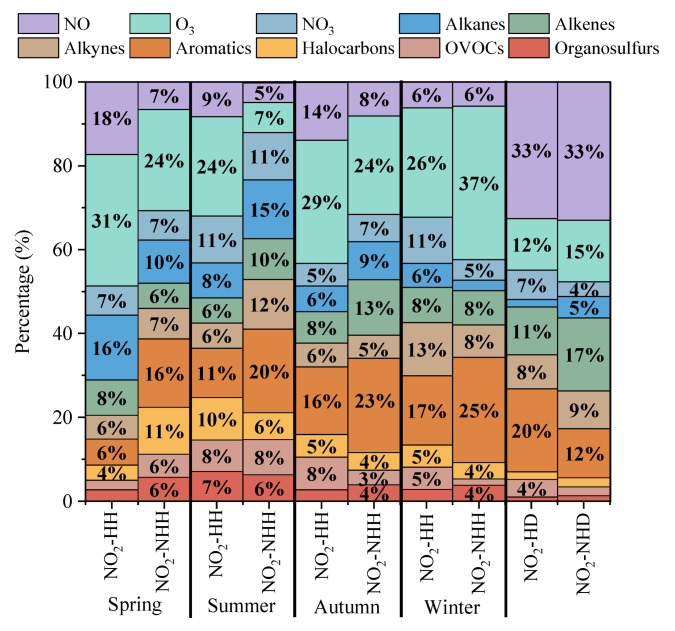
The importance share of each pollutant’s influence on NO_2_ concentrations on days with NO_2_-HH events and NO_2_-NHH periods in each season of Dongying in 2022.

The factor importance obtained after building a random forest model based on pollutant data is shown in Figure 18. Overall, the main pollutants influencing the hourly NO_2_ concentrations in Dongying City were NO, O_3_, and Aromatics, while the dominant factors for the daily NO_2_ concentrations were NO, Aromatics, Alkenes, and O_3_. From the difference between NO_2_-HH and NO_2_-NHH, the importance of O_3_ and NO increased for NO_2_-HH, except in winter, while the importance of Aromatics decreased across all seasons. Other pollutants showed no consistent pattern. This suggests that NO_2_-HH in spring, summer, and autumn is primarily driven by the titration reaction between NO and O_3_, while the day–night variation in Aromatics is related to the concentrations of oxidants OH and NO_3_ [68]. The weakening of reactions with NO_3_ reduces the net consumption of NO_2_. From the difference between NO_2_-HD and NO_2_-NHD, the importance of Alkenes decreased, while that of Aromatics increased. This is because Alkenes are key participants in the nighttime chemical reaction chain, consuming NO_3_ and leading to net consumption of NO_2_ [69]. Both Alkenes and Aromatics exhibit strong photochemical activity during the day, participating in photochemical chain reactions to produce NO_2_ [70].

Overall, daytime NO_2_ concentration are primarily influenced by intense photochemical reactions, with higher VOCs and NO concentrations leading to higher daytime NO_2_ levels. During nighttime and morning rush hours, NO and O_3_ significantly affect NO_2_ concentration. The titration reaction between NO with O_3_ is the main cause for NO_2_-HH in spring, summer and autumn, and photochemical reactions of Aromatics have a significant influence on NO_2_-HD.

#### 3.3.3. Sources Analysis

**Figure 19:**
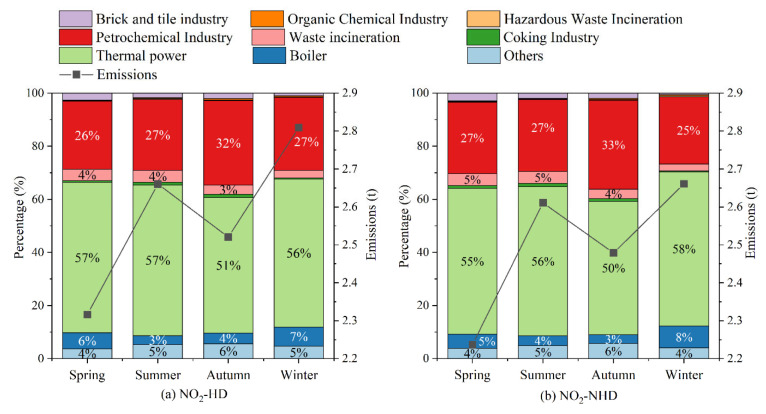
Differences in emissions and percentages of NOx from different industry between NO_2_-HD days and NO_2_-NHD days for each season in Dongying in 2022.

As shown in Figure 19, NOx emissions in Dongying are generally higher on NO_2_-HD days compared to NO_2_-NHD days across all seasons, with the greatest difference in winter and the smallest in summer. This indicates that local NOx emissions have a significant impact on NO_2_-HD in Dongying. Seasonal variations in NOx emissions are highest in winter and lowest in spring, which differs from the seasonal variations in NO_2_ concentration, suggesting that NO_2_ distribution may be influenced by additional factors. Industry-specific NOx emissions data reveal that thermal power (51–57%) and petrochemical industry (26–32%) are the largest contributors to NOx emissions across all seasons. Compared to NO_2_-NHD days, on NO_2_-HD days, the proportion of emissions from thermal power increases in spring, summer, and autumn, while the proportion from the petrochemical industry increases in winter. Therefore, thermal power and petrochemical industry are likely major sources affecting NO_2_-HD. In summary, local emissions (thermal power and petrochemical industry) have a certain impact on NO_2_-HD in Dongying.

**Figure 20:**
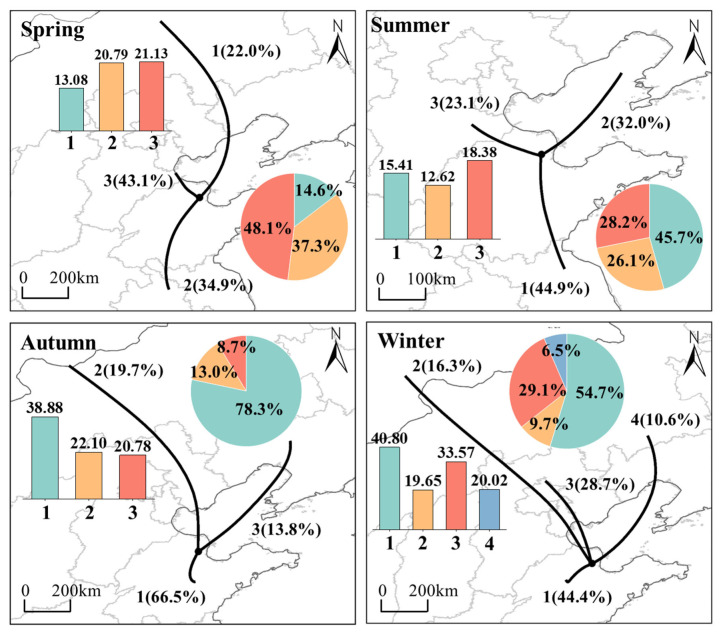
Cluster analysis of backward trajectories in Dongying in 2022. (The bar chart represents the average NO_2_ concentration (μg/m^3^) corresponding to each type of trajectory, and the pie chart represents the percentage contribution of NO_2_ concentration corresponding to each type of trajectory).

Cluster analysis (Figure 20 and Figure S12) reveals significant seasonal differences in the direction, speed, and altitude of air masses at Dongying sites. In spring, northwest trajectory 3 accounts for 43.1%, with the shortest trajectory, indicating slower air mass movement and a higher potential for local pollutant accumulation. This trajectory also operates at a lower altitude, facilitating local horizontal pollutant transport. The corresponding average NO_2_ concentration for this trajectory was 21.1 μg/m^3^, contributing 48.1%. Southwest trajectory 2, which accounts for 34.9%, has a relatively short trajectory and the lowest altitude, leading to pollutant transport from southern cities in Shandong Province. Additionally, trajectory 1, with a lower proportion, has a higher altitude and exerts minimal impact on Dongying in spring. In summer, the southeastward trajectory had the highest share (44.9%) and contribution (45.7%). The average concentration for this trajectory matched the summer average NO_2_ concentration, indicating that this trajectory is the primary source of air masses maintaining summer NO_2_ levels. Trajectory 3 had the lowest share (23.1%) but the highest average concentration (18.4 μg/m^3^), which facilitates the formation of high NO_2_ values. Additionally, Dongying City in summer may be affected by the transport of air masses from the Bohai Bay (32%). Similarly, in autumn and winter, the local influence on Dongying City is significantly stronger, primarily from air masses coming from the south and southwest. This is related to air mass transport and pollutant accumulation [71]. The lifespan of NO_2_ is relatively short (1–2 days), primarily influenced by local transport rather than long-distance transport [72].

**Figure 21:**
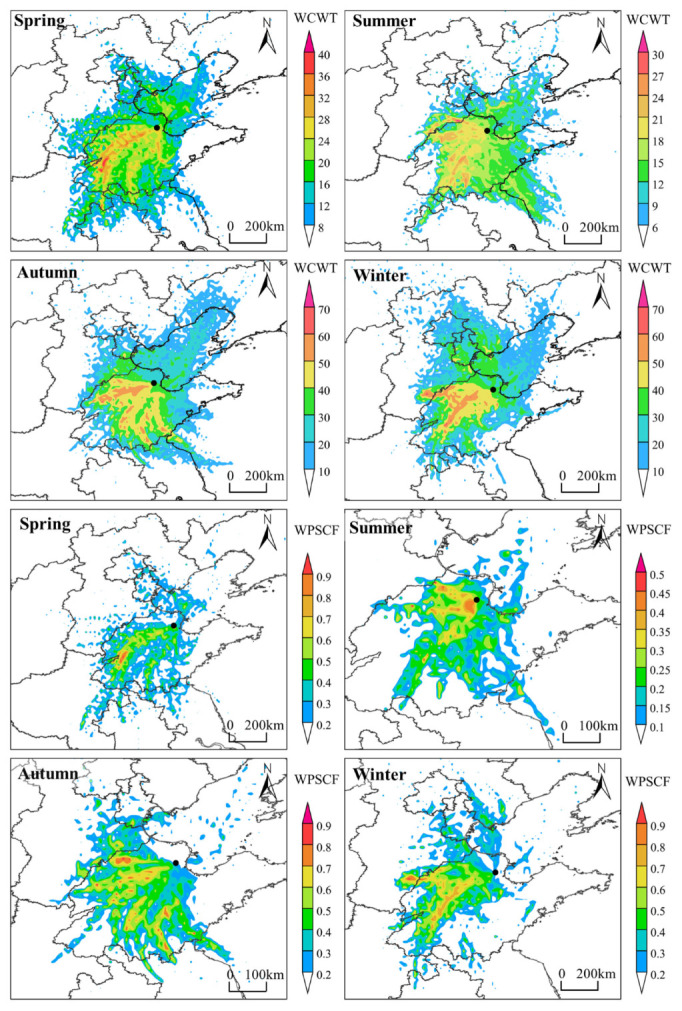
Distribution of WPSCF and WCWT in Dongying in 2022 (The black dot represents Dongying Atmospheric Observatory).

The distribution of WPSCF and WCWT in Dongying in 2022 is shown in Figure 21. From the WPSCF distribution, in spring, the WPSCF values for the southwestern regions of Dongying and the small area around the Bohai are relatively high, with a focus on cities in western Shandong Province, such as Jinan, Binzhou, and Taian, with western Taian having the highest potential impact on NO_2_-HH. In summer, the WPSCF values for nearby cities of Dongying, particularly Binzhou and Zibo, are higher, indicating a significant influence from nearby cities. In autumn, potential source regions affecting NO_2_-HH in Dongying are more widely distributed, primarily in central Dezhou, Jinan, Taian, and parts of Linyi. In winter, the main potential source regions affecting NO_2_-HH in Dongying are located in northeastern Handan, southwestern Dezhou, and central areas of Jinan and Taian. From the WCWT distribution, the distribution of WCWT values is quite similar to that of WPSCF values, with the areas of high concentration contribution aligning well with the potential source regions that significantly impact NO_2_-HH at the observation site. In spring, autumn, and winter, areas with WPSCF values greater than 0.5 generally correspond to areas with high WCWT values, while in summer, areas with WPSCF values greater than 0.25 align with areas of high WCWT values. This correspondence is related to the NO_2_ threshold settings in the seasonal PSCF analysis. In addition to the regions corresponding to the high WPSCF values, there are some areas with high WCWT values in the Bohai Sea of China during spring and summer and in the northwest of Dongying during winter.

In summary, the distribution of high WCWT and WPSCF values is quite similar and aligns closely with the main air mass transport pathways. NO_2_ regional transport in Dongying primarily occurs within Shandong Province. The potential source regions that contribute most significantly to the occurrence probability and concentration of NO_2_-HH at the Dongying site are mainly located in the western and southwestern parts of Shandong Province (with wider distribution in autumn and winter), including Jinan, Binzhou, Taian, and Zibo. Additionally, during spring and summer, there is a certain influence from the Bohai Sea of China.

Dongying City is located in the Bohai Bay region, which has a unique geographical location and meteorological conditions [19,55]. In the future, building on outstanding international research, it is highly necessary to conduct long-term and large-scale studies on NO_2_ to provide scientific support for regional NO_2_ control and air quality improvement.

## 4. Conclusions

In this study, we explored the effects of NO_2_ on the ambient air quality in Dongying, a typical petrochemical city in Bohai Bay of China, from 2017 to 2023 and the impacts of high NO_2_ values on the concentrations of O_3_, PM_2.5_ and PM_2.5_-bounded secondary components, and atmospheric oxidation capacity in 2022, and investigated the reasons for the formation of high NO_2_ values in Dongying.

(1) From 2017 to 2023, the annual assessment values of NO_2_ concentrations in Dongying exhibited a trend of initially decreasing and then stabilizing, with monthly variation characterized by a “U” distribution, diurnal variation characterized by a “single-peak, single-valley” distribution, and spatial variation characterized by a “high in the south-central part and low in the north” distribution. Higher daily NO_2_ concentrations mainly occurred during January-April and September-December each year, and higher hourly NO_2_ concentrations mainly occurred during the nighttime and morning rush hours.
(2) NO_2_ is a contributing factor to the increase in various pollutant concentrations, thereby affecting air quality levels. High hourly NO_2_ values (NO_2_-HH) events promoted the increase in the concentrations of PM_2.5_ and PM_2.5_-bounded NO_3_^-^, SO_4_^2-^ and SOC, and O_X_ during the nighttime and morning rush hours, and they also facilitated the increase in the concentrations of O_3_, PM_2.5_ and PM_2.5_-bounded NO_3_^-^, SO_4_^2-^ and SOC, and O_X_, as well as NOR and SOR values during the daytime. High daily NO_2_ values (NO_2_-HD) could promote the increase in the concentrations of O_3_ (MDA8-O_3_), PM_2.5_ and PM_2.5_-bounded NO_3_^-^, SO_4_^2-^ and SOC, and O_X_ on the same day.
(3) The occurrence of high NO_2_ values was affected by the combination of unfavorable meteorological conditions, local emissions and regional air pollution transports, and localized atmospheric chemical generation. High-pressure and uniform-pressure weather patterns, along with low temperatures (−5~15 °C), moderate humidity (30~80%), wind speeds below 1 m/s, and southwesterly winds, are conducive to high daily NO_2_ values. In 2022, land–sea breeze circulation significantly contributed to high NO_2_ concentrations. Random forest analysis indicates that in 2022, BLH in spring (−0.43) and winter (−0.36), WD in summer (0.21), and T (−0.46) in autumn were the primary factors contributing to NO_2_-HH, while BLH (−0.47) is the main cause for NO_2_-HD. The titration reaction between NO with O_3_ is the main cause for NO_2_-HH in spring, summer and fall, and photochemical reactions of Aromatics have a significant influence on NO_2_-HD. NOx emissions from the thermal power and petrochemical industry in Dongying and air pollution transports from western and southwestern Shandong Province (throughout the year) and from the Bohai Sea (during spring and summer) had serious adverse impact on high NO_2_ values.

It is recommended that Dongying strengthens its control over NO_X_ emissions from local thermal power and petrochemical industries, and actively promotes the establishment of an air pollution prevention and control mechanism in collaboration with cities in western and southwestern Shandong Province and the Bohai Bay region of China to effectively reduce NO_2_ concentrations and prevent high NO_2_ values, thereby continuously improving air quality.

## Figures and Tables

**Table 1:** Frequency and major characteristics of nine synoptic patterns of the 925-hPa geopotential height field in Dongying from 2017 to 2022.

Synoptic Pattern	Frequency	Major Characteristics
Type1	20.5%	Dongying near the offshore high-pressure rear, with dominant southeasterly wind, T_max_-mean of 21.8 °C, RH-mean of 56.6%, and Pre-mean of 1.4 mm.
Type2	14.1%	High-pressure center over the Mongolian Plateau. Dongying under the front of the high-pressure, with dominant southeasterly wind, T_max_-mean of 10.4 °C, RH-mean of 53.9%, and Pre-mean of 0.8 mm.
Type3	12.1%	High-pressure center over the North China Plain. Dongying inside the high-pressure, with dominant easterly wind, T_max_-mean of 16.9 °C, RH-mean of 64.0%, and Pre-mean of 1.4 mm.
Type4	16.6%	High-pressure center over the North China Plain. Dongying under the top-front of the high-pressure, with dominant southwesterly wind, T_max_-mean of 14.5 °C, RH-mean of 57.0%, and Pre-mean of 0.9 mm.
Type5	6.7%	Dongying under the control of a weak pressure field with sparse isobars and stable synoptic condition, with dominant southerly wind, T_max_-mean of 17.3 °C, RH-mean of 64.0%, and Pre-mean of 1.9 mm.
Type6	8.4%	Dongying between a high- and low-pressure systems with dense isobars, with dominant southeasterly and southwesterly winds, T_max_-mean of 19.2 °C, RH-mean of 56.8%, and Pre-mean of 0.6 mm.
Type7	9.5%	Dongying in the northwest of the western Pacific subtropical high, with dominant southeasterly wind, T_max_-mean of 29.2 °C, RH-mean of 72.4%, and Pre-mean of 8.0 mm.
Type8	7.6%	Dongying near the inverted trough, with dominant southeasterly wind, T_max_-mean of 25.8 °C, RH-mean of 73.2%, and Pre-mean of 6.6 mm.
Type9	4.4%	Dongying inside the low-pressure field, with dominant southeasterly wind, T_max_-mean of 29.3 °C, RH-mean of 69.1%, and Pre-mean of 2.1 mm.

Notes: T_max_-mean refers to the daily average maximum temperature; RH-mean refers to the daily average relative humidity; and Pre-mean refers to the daily average precipitation.

## Data Availability

The raw data supporting the conclusions of this article will be made available by the authors on request.

## Supplementary Materials

The following supporting information can be downloaded at: https://www.mdpi.com/article/10.3390/toxics13030208/s1, Figure S1: Conceptual diagram of NO_2_ sources, sinks, and impacts; Figure S2: Box plots of daily average concentrations (a) and hourly concentrations (b) of NO_2_ in Dongying for each season in 2022; Figure S3: Technical roadmap of analyzing the impact of NO_2_ on air quality in Dongying; Figure S4: Technical roadmap of analyzing the causes of high NO_2_ values in Dongying in 2022; Figure S5: Comparison of diurnal variations of NO_2_ concentrations in Dongying from 2017 to 2023; Figure S6: Percentages of days with different NO_2_ air quality levels and number of days with NO_2_ as the primary pollutant in Dongying from 2017 to 2023; Figure S7: Comparison of monthly distribution of NO_2_-HD days in Dongying in 2022; Figure S8: Comparison of seasonal distribution of NO_2_-HH in Dongying in 2022; Figure S9: Differences in the daytime concentrations of O_3_, PM_2.5_ and PM_2.5_-bounded secondary components between NO_2_-HH events and NO_2_-NHH periods in Dongying in 2022; Figure S10: Differences in the daytime concentrations of O_X_, and values of NOR and SOR between NO_2_-HH events and NO_2_-NHH periods in Dongying in 2022; Figure S11: Classification of 925-hPa synoptic patterns in Dongying from 2017 to 2022; Figure S12: Mean barometric pressure variation curves in the vertical direction during the movement of clustered trajectories in each season in 2022; Text S1: Data sources; Text S2: Data quality control; Text S3: Synoptic classification method; Text S4: HYSPLIT mode; Text S5: Backward trajectory clustering analysis; Text S6: PSCF analysis; Text S7: CWT analysis; Text S8: Analysis of impact of high NO_2_ values on daytime O_3_, PM_2.5_, and atmospheric oxidation capacity in Dongying in 2022; Table S1: Data types and data sources for each part of analysis in this study; Table S2: Correlation between NO_2_ concentration and meteorological factors in different seasons in Dongying from 2017 to 2022 [73,74,75,76,77,78,79,80,81,82,83,84,85,86].
