# Supplementary material for: Impacts of NO2 on Urban Air Quality and Causes of Its High Ambient Levels: Insights from a Relatively Long-Term Data Analysis in a Typical Petrochemical City in the Bohai Bay Region, China
Source: Toxics. 2025 Mar 13;13(3):208. doi: 10.3390/toxics13030208 (PMC11946341; doi:10.3390/toxics13030208)
Supplement: Supplementary file 1 [file toxics-13-00208-s001.zip › toxics-3500070-supplementary.pdf]

Supplement of

The Impacts of NO2 on Urban Air Quality and Cause for the Investigation of Its High Ambient Levels: Insights from a Relatively Long-Term Data Analysis in a Typical Petrochemical City in the Bohai Bay Region, China

Xiaoshuai Gao 1,2, Cong An 3, Yongxin Yan 1, Yuanyuan Ji 1, Wei Wei 2, Likun Xue 4, Rui Gao 1 , Fanyi Shang 5, Jidong Li 5, Luyao Tan 6 and Hong Li 1,*

1 State Key Laboratory of Environmental Criteria and Risk Assessment, Chinese Research Academy of Environmental Sciences, Beijing 100012, China; s202265286@emails.bjut.edu.cn (X.G.); yyx_in@163.com (Y.Y.); ji.yuanyuan@craes.org.cn (Y.J.); gaorui@craes.org.cn (R.G.)

2 Department of Environmental Science and Engineering, Beijing University of Technology, Beijing 100124, China; weiwei@bjut.edu.cn

3 Shanghai Key Laboratory of Atmospheric Particle Pollution and Prevention, Department of Environmental Science and Engineering, Fudan University, Shanghai 200438, China; ancong_22@163.com

4 Environment Research Institute, Shandong University, Qingdao 266237, China; xuelikun@sdu.edu.cn

5 Dongying Municipal Ecology and Environment Bureau, Dongying 257000, China; dyhbsfy@126.com (F.S.); shbjzlb@dy.shandong.cn (J.L.)

6 Ltd. of Shandong Environmental Protection Industry Corp., Jinan 250061, China; tanluyyy@163.com

* Correspondence: lihong@craes.org.cn

1. Figures

1.1. **Figure S1.**

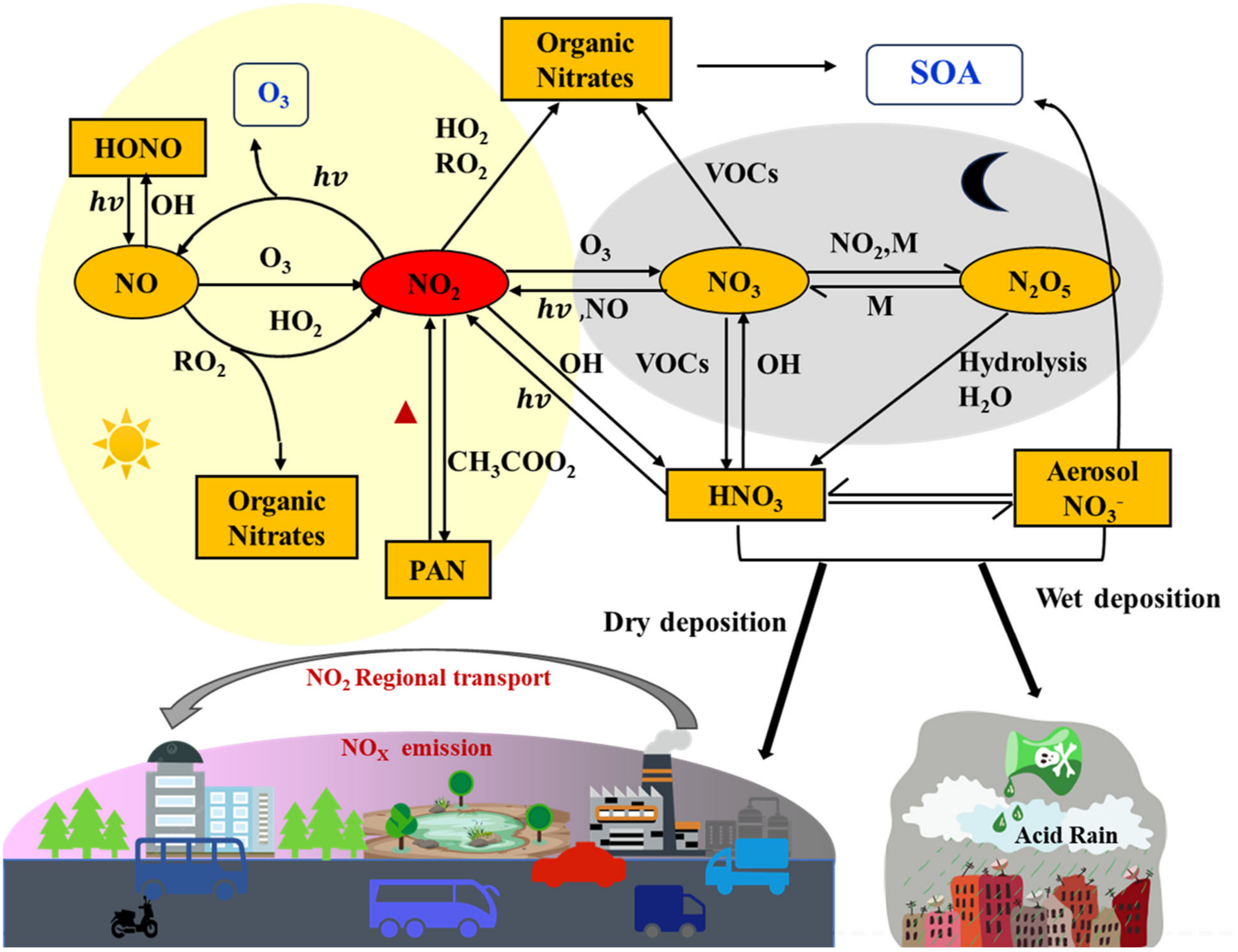

Figure S1. Conceptual diagram of NO2 sources, sinks, and impacts

1.2. **Figure S2.**

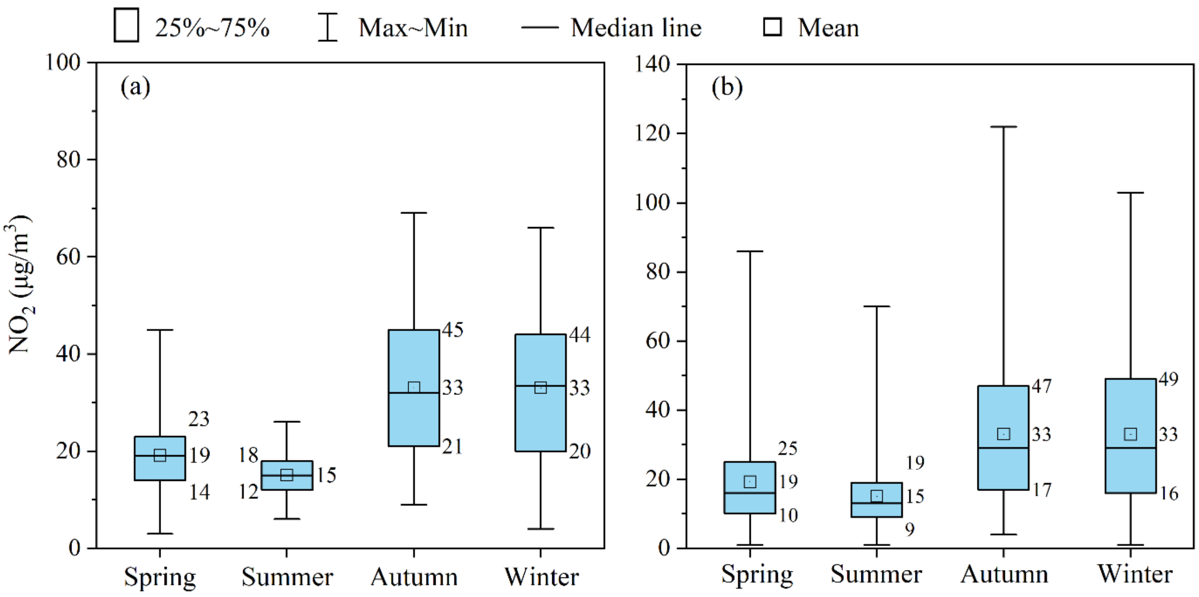

Figure S2. Box plots of daily average concentrations (**a**) and hourly concentrations (**b**) of NO_2 in Dongying for each season in 2022

1.3. **Figure S3.**

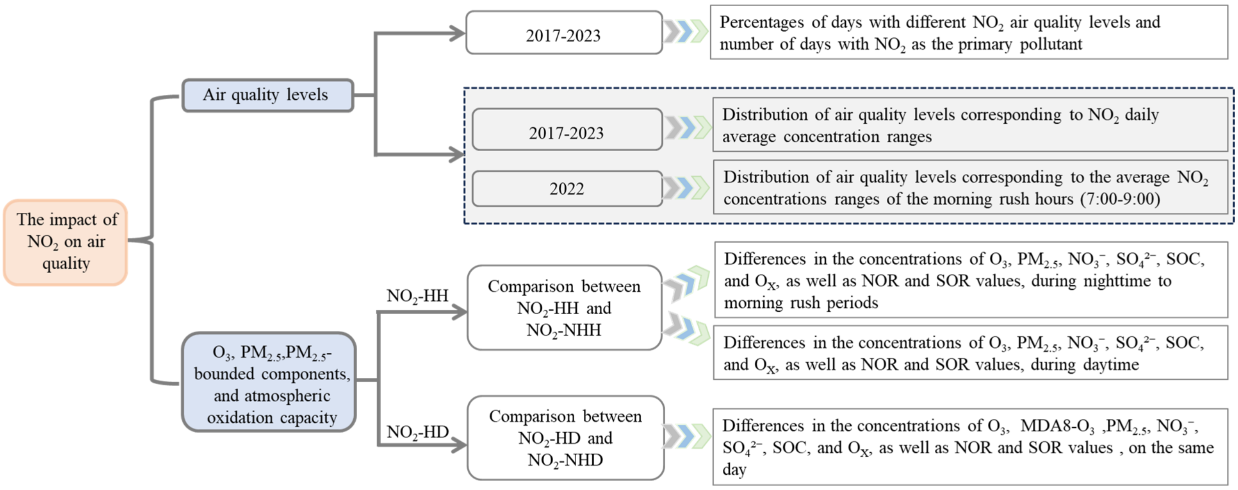

Figure S3. Technical roadmap of analyzing the impact of NO2 on air quality in Dongying.

1.4. **Figure S4.**

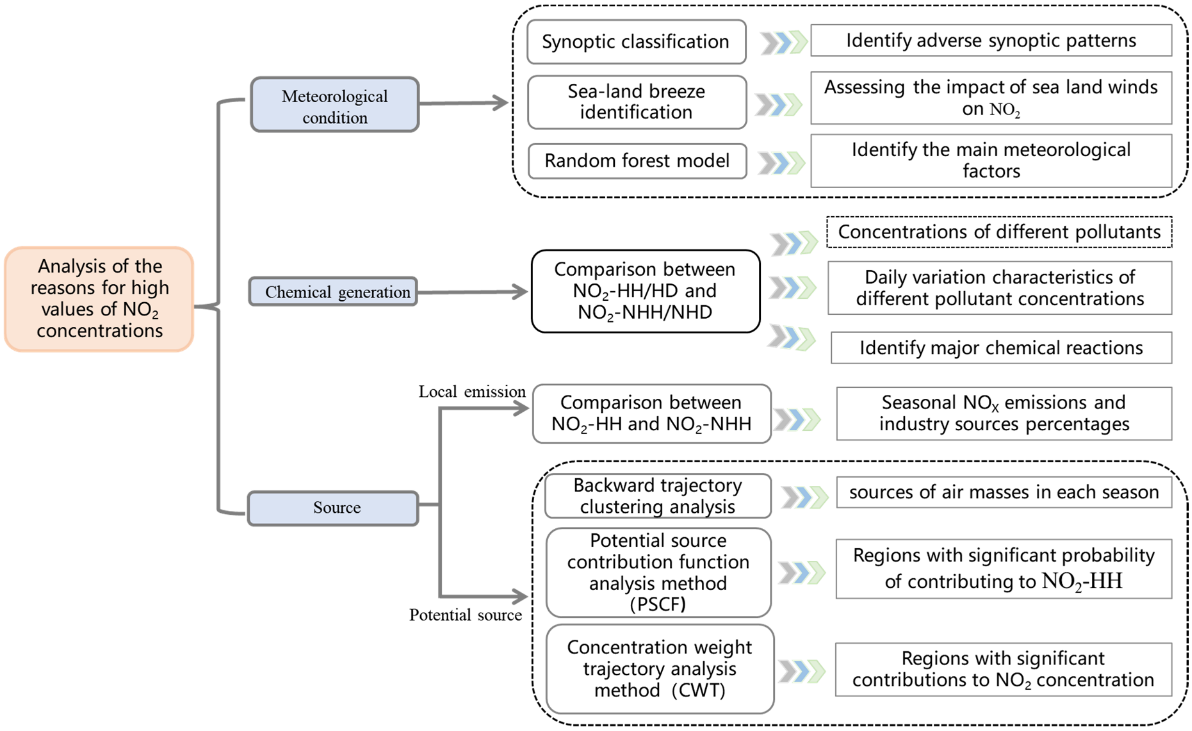

Figure S4. Technical roadmap of analyzing the causes of high NO_2 values in Dongying in 2022.

1.5. **Figure S5.**

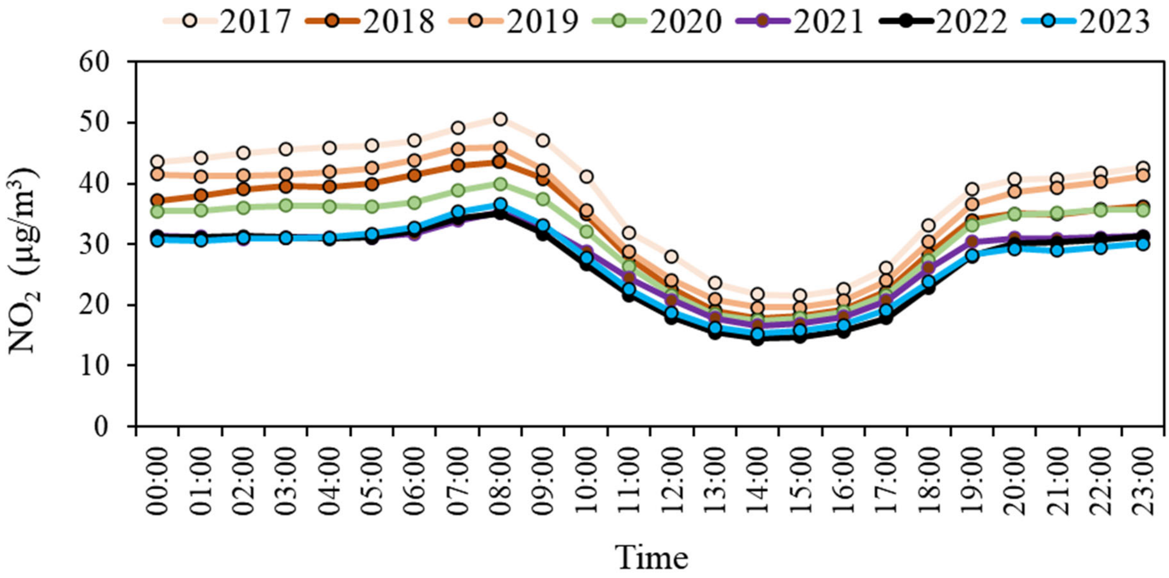

Figure S5. Comparison of diurnal variations of NO_2 concentrations in Dongying from 2017 to 2023

1.6. **Figure S6.**

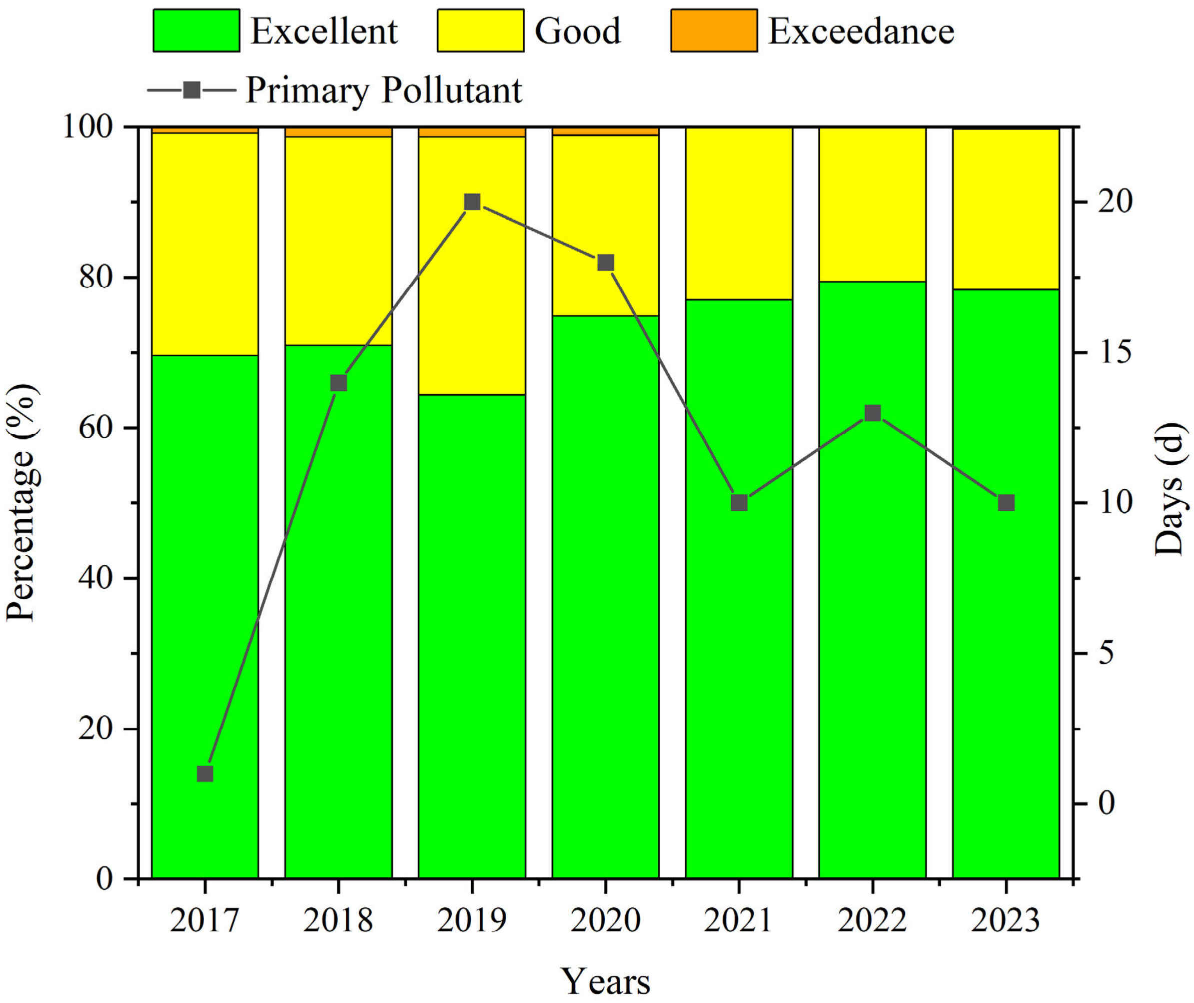

Figure S6. Percentages of days with different NO_2 air quality levels and number of days with NO_2 as the primary pollutant in Dongying from 2017 to 2023

1.7. **Figure S7.**

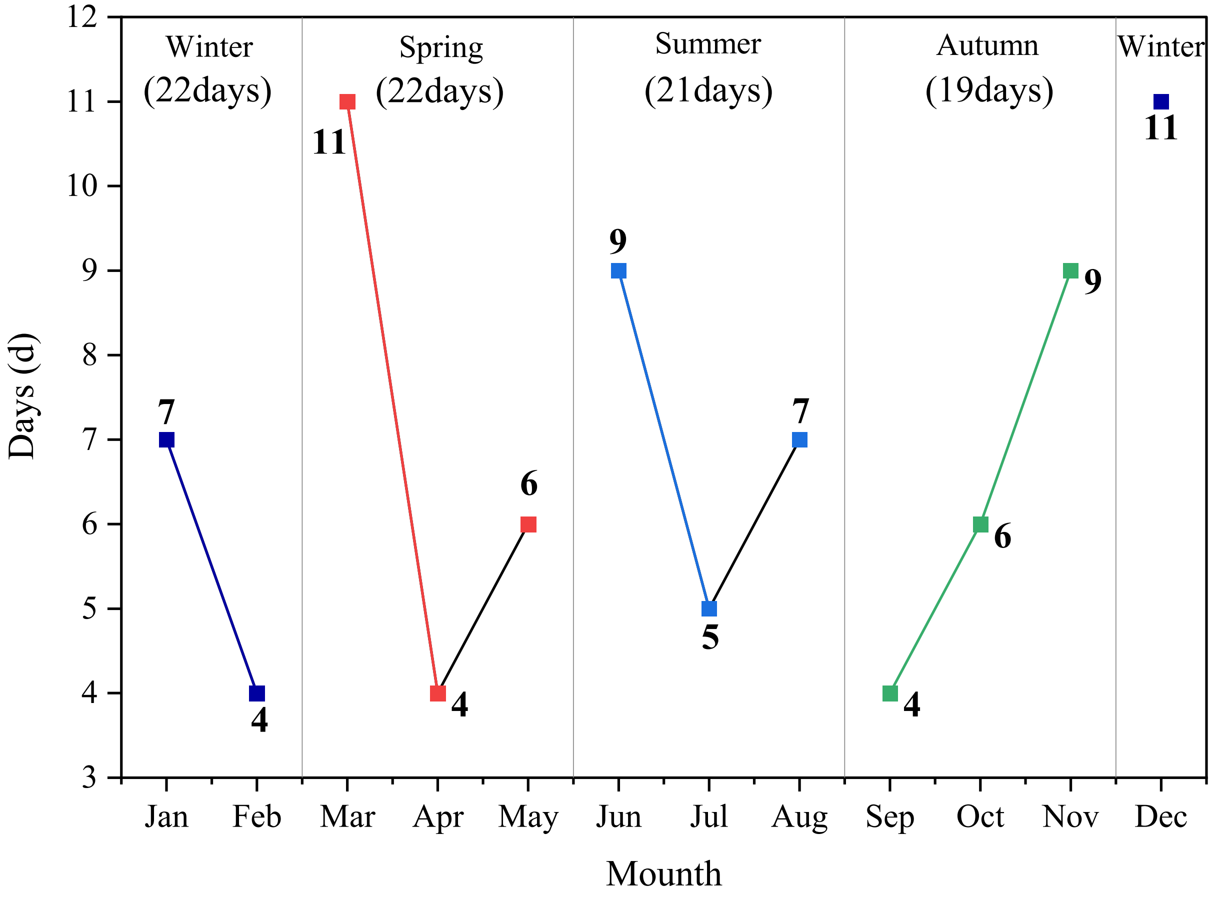

Figure S7. Comparison of monthly distribution of NO_2-HD days in Dongying in 2022

1.8. **Figure S8.**

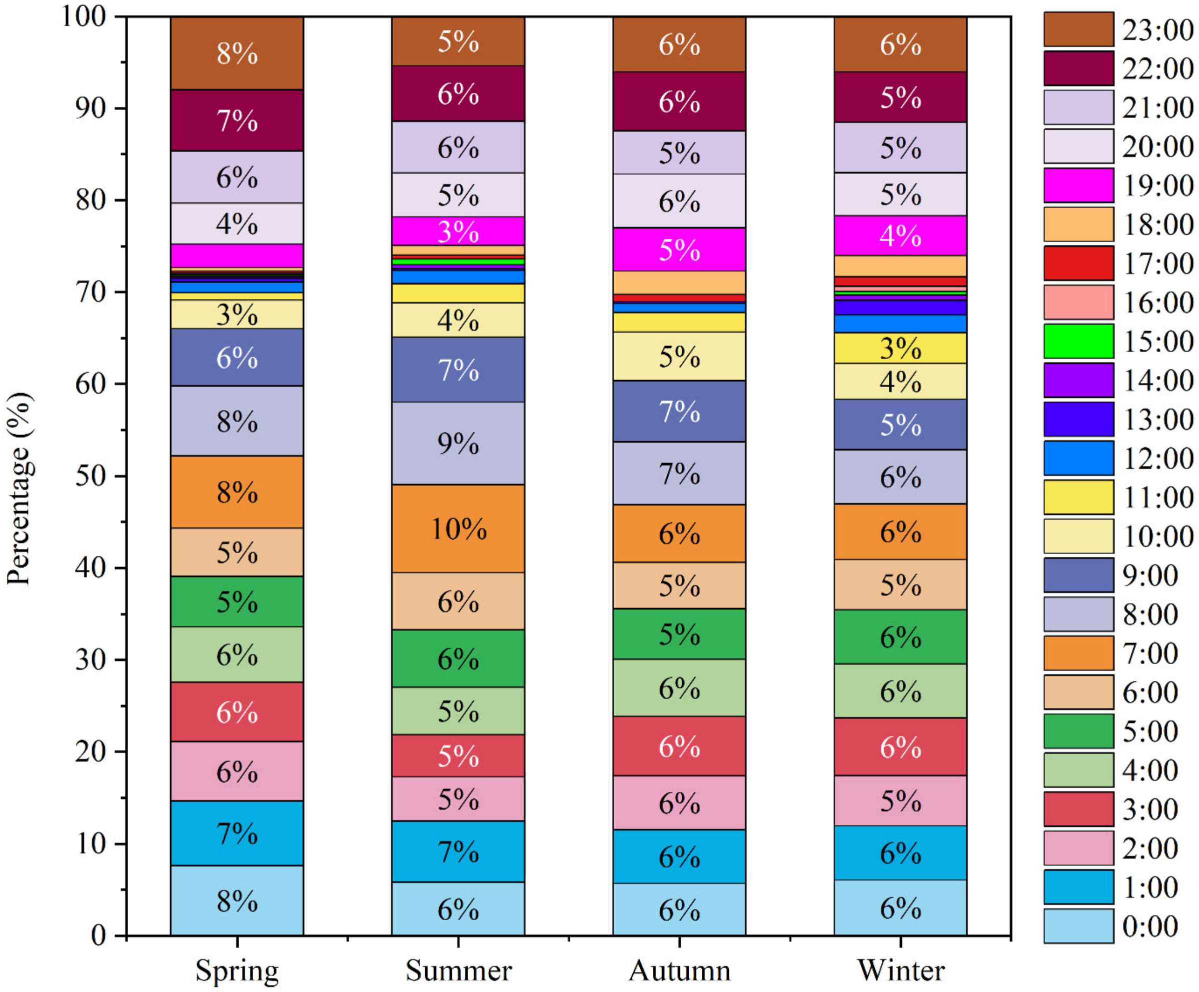

Figure S8. Comparison of seasonal distribution of NO_2-HH in Dongying in 2022

1.9. **Figure S9.**

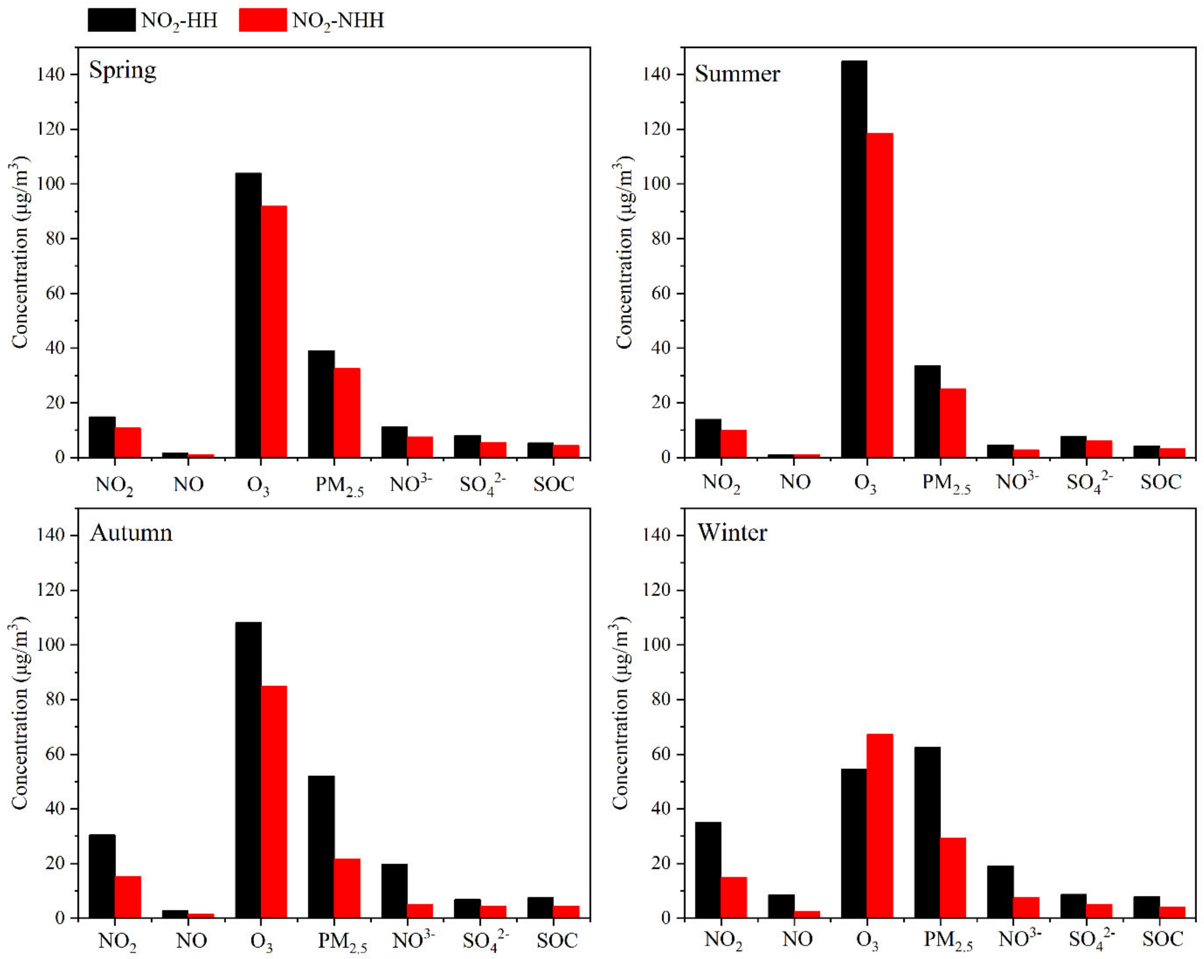

Figure S9. Differences in the daytime concentrations of O_3, $PM_{2.5}$ and $PM_{2.5}$-bounded secondary components between NO_2-HH events and NO_2-NHH periods in Dongying in 2022

1.10. **Figure S10.**

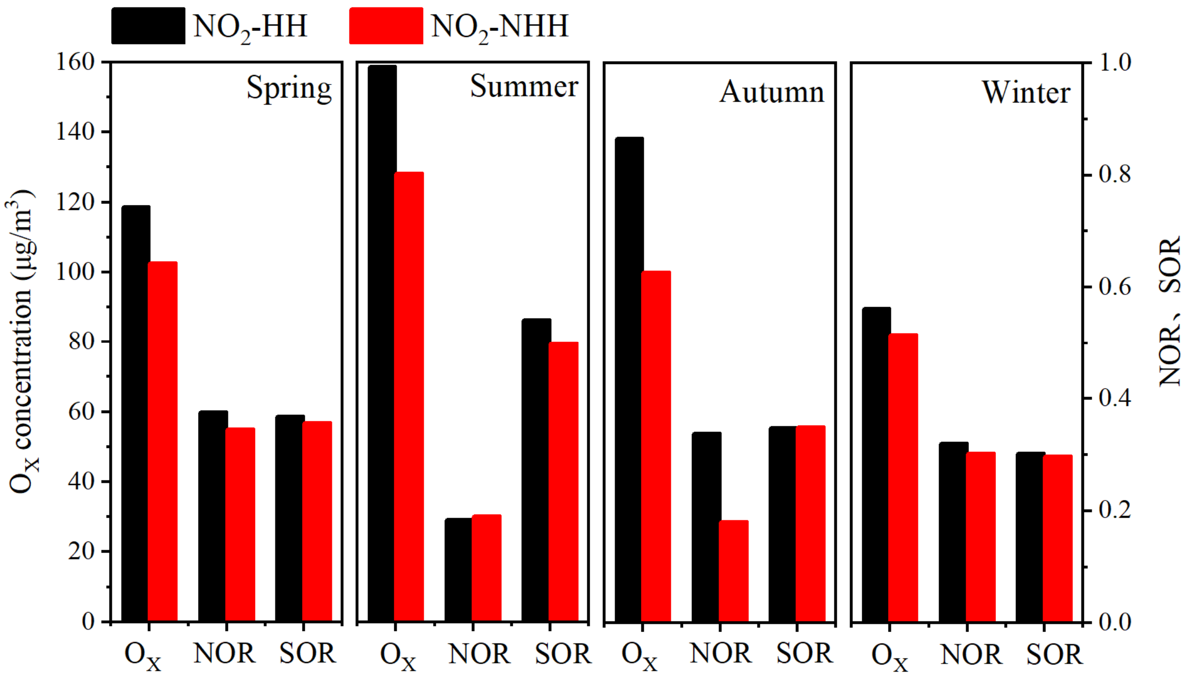

Figure S10. Differences in the daytime concentrations of O_X, and values of NOR and SOR between NO_2-HH events and NO_2-NHH periods in Dongying in 2022

1.11. **Figure S11.**

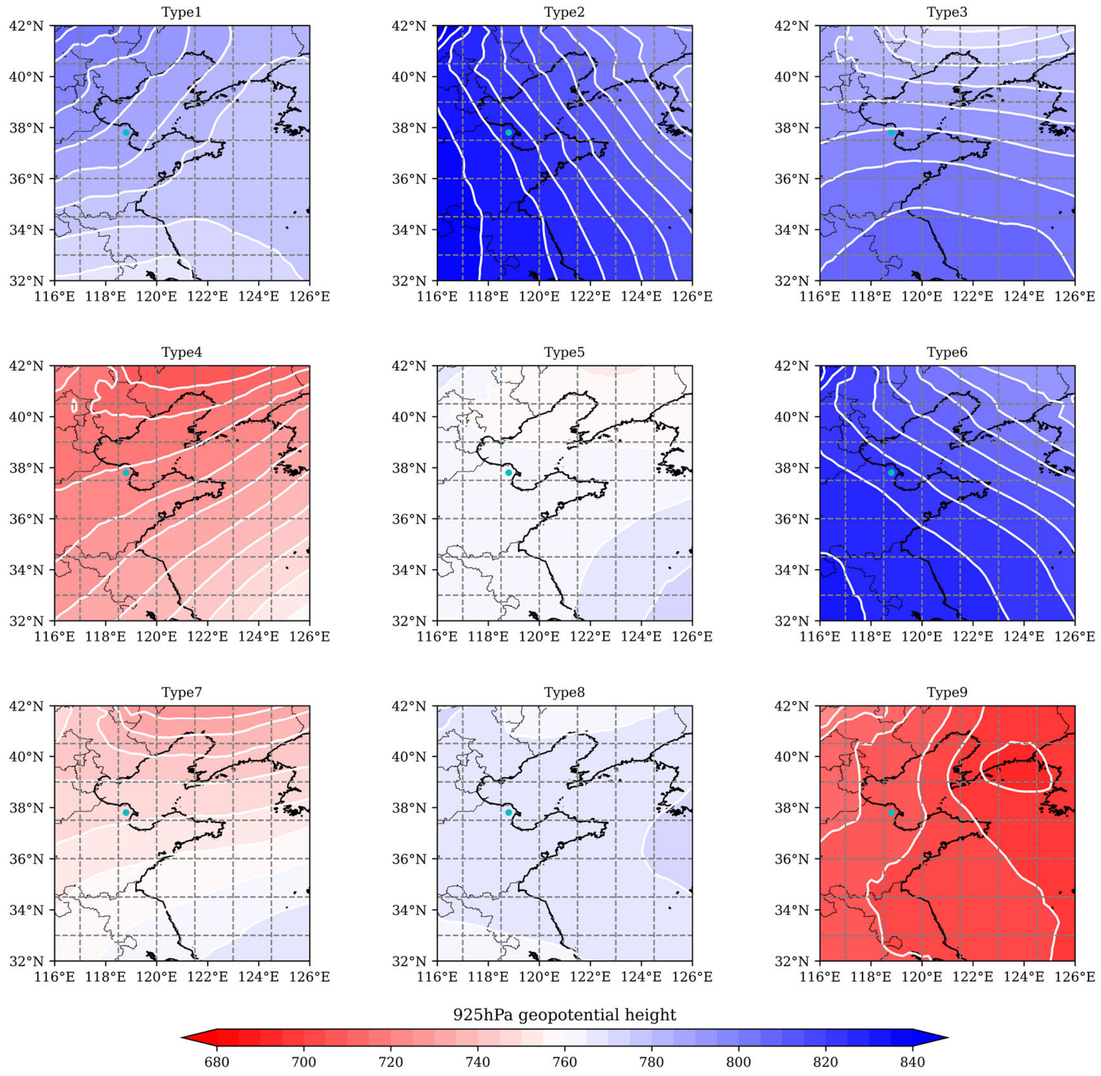

Figure S11. Classification of 925-hPa synoptic patterns in Dongying from 2017 to 2022

1.12. **Fig**ure **S12.**

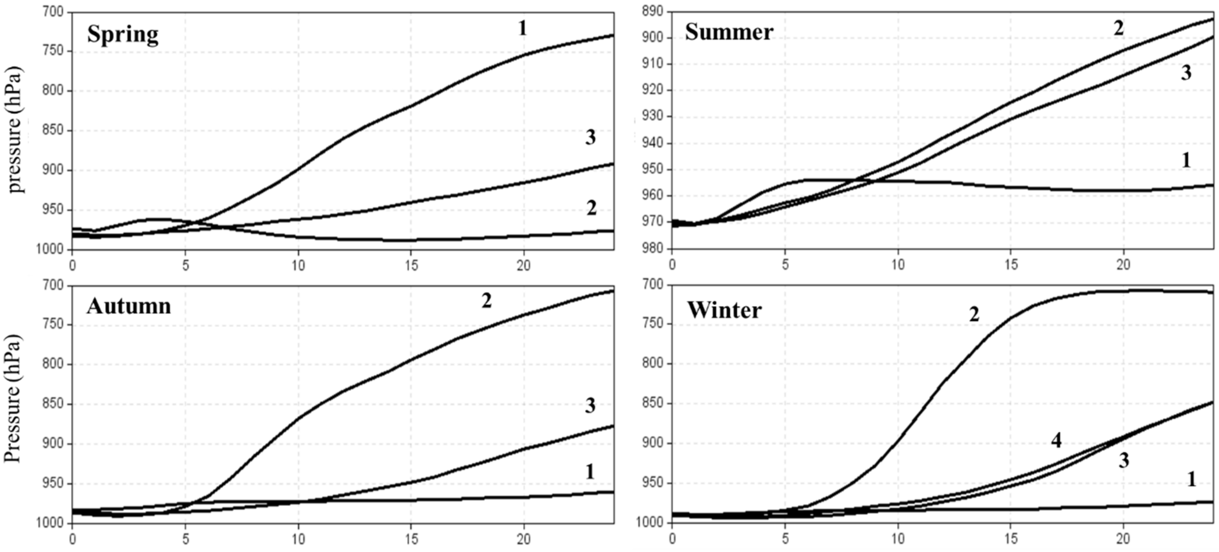

Figure **S12.** Mean barometric pressure variation curves in the vertical direction during the movement of clustered trajectories in each season in 2022.

2. Texts

2.1. **Text. S1. Data sources**

All observation items were continuously monitored with automatic monitoring devices. SO_2, CO, NO, NO_2, NOx, and O_3 were monitored with 43i, 48i, 42i, and 49i (Thermo Fisher Scientific Inc., WA, Massachusetts, USA), respectively. Particulate matters were monitored online with BAM1020 (Met One Instruments Inc., Washington, DC, USA). VOCs were monitored with XHVOC6000 (Hebei Sailhero Environmental Protection Hi-tech., Ltd., Shijiazhuang, Hebei, China). The OC and EC analyzer model was OCEC-100 (Focused Photonics Inc., Hangzhou, Zhejiang, China). The elemental analyzer model was CES Xcat625 (Sailbri Cooper Inc., Tigard, OR, USA). The ion component analyzer model was S−611EG (Zhang Jia Ltd., Taiwan, China). The ultraviolet radiation observer model was Kipp and Zonen SUV-A, SUV-B (OTT HydroMet B.V., Delft, The Netherlands). The meteorological parameters were obtained from the Dongying National Basic Meteorological Station (elevation 6m, 118.40°E, 37.26°N). The emission source data were obtained from Dongying environmental automatic monitoring and control system.

2.2. **Text. S2. Data quality control**

The frequency and duration of data collection, quality assurance, and quality control of all observation items met the requirements of the technical specifications such as Automated Methods for Ambient Air Quality Monitoring (HJ/T193-2005), Specifications and Test Procedures for Ambient Air Quality Continuous Monitoring System with Gas Chromatography for Volatile Organic Compounds (HJ 1010-2018), Technical Specifications for Continuous Automated Monitoring of Organic carbon and Elemental Carbon in Ambient Air Particulate Matter ($PM_{2.5}$) (Draft), Technical Specifications for Continuous Automated Monitoring of Water-Soluble Ions in Ambient Air Particulate Matter ($PM_{2.5}$) (Draft), and Technical Specifications for Continuous Automated Monitoring of Inorganic Elements in Ambient Air Particulate Matter ($PM_{2.5}$) (Draft) The values recorded by the devices could be calculated as hourly arithmetic means. The monitoring station complies with standards such as the Technical Specifications for Operation and Quality Control of Ambient Air Quality Continuous Automated Monitoring System for SO_2, NO_2, O_3; and CO (HJ 818-2018), Technical Requirements on Site Selection of National Meteorological Observing Station (QX/T 684-2023), and Specifications for Surface Meteorological Observation—General (HJ 35221-2017), and meets the requirements of the World Meteorological Organization (WMO), ensuring the accuracy and consistency of the observational data.

Additionally, data quality control in this study was performed in accordance with the Ambient Air Quality Standards (GB 3095-2012) [1]., the Technical Regulation for Ambient Air Quality Assessment (on Trial) (HJ 663-2013) [2], and the Technical Regulation on Ambient Air Quality Index (on Trial) (HJ 633-2012) [3]. Missing and abnormal values were excluded.

2.3. **Text. S3. Synoptic classification method**

T-mode Principal Component Analysis (PCT) basic principle involves calculating the eigenvectors of the original data matrix Z, forming the loading matrix A with these eigenvectors, and performing an oblique rotation of the principal component matrix F based on the largest eigenvalues, i.e., Z = FAT. The weather classification for each time step is then determined based on the size of the loadings [4]. Multivariate oblique rotation decomposition of the 925 hPa geopotential height field and horizontal full WS (U and V) was performed to reveal the spatial distribution of variables and the spatial relationships among them, leading to more accurate circulation classification results [5]. Each circulation type was named based on the location of the surface weather system in Dongying.

Meteorological reanalysis data used for objective synoptic classification were sourced from the European Centre for Medium-Range Weather Forecasts (ECMWF) ERA5 dataset, with a resolution of 0.25°×0.25°. Considering that the 08:00 (BJT) reanalysis data assimilate global surface and sounding observations, providing the highest accuracy and better reflecting the actual conditions compared to data from other times, this study selected the 08:00 reanalysis data for weather classification analysis. To minimize the influence of near-surface friction on the vector wind field, this study selected the 925 hPa geopotential height field and the horizontal wind components (U and V) for classification. The study area extends from 32° to 42°N and 114° to 126°E, including Dongying City and the surrounding continental and marine regions.

2.4. **Text. S4. HYSPLIT mode**

The Hybrid Single-Particle Lagrangian Integrated Trajectory Model (HYSPLIT) is primarily used for analyzing air parcel trajectories. Developed by NOAA's Air Resources Laboratory [6], it utilizes Meteoinfo software and the TrajStat plugin to visualize GDAS data, enabling the analysis of atmospheric pollution transport pathways and potential source areas for a specific region [7]. GDAS data, provided by the National Environmental Prediction Center of the United States, includes meteorological information such as T, pressure, and WS. The analysis of transport pathways and potential pollution source areas is based on a hybrid Eulerian-Lagrangian dispersion model, which effectively tracks the movement of pollutants carried by airflows [8]. This model considers both local sources and the influence of surrounding regions [9]. Considering factors such as Dongying City's elevation, NO_2 vertical distribution characteristics, atmospheric lifetime, and trajectory model resolution, the analysis height for air parcels was set at 300 meters [10], with a backward tracing time of 24 hours.

2.5. **Text. S5. Backward trajectory clustering analysis**

Backward trajectory clustering analysis uses the clustering analysis function in the TrajStat plugin, all air parcel trajectories are subjected to Eulerian clustering analysis. The number of clustered trajectories is determined by comprehensively judging the TSV diagram and the actual pollution air mass conditions in Dongying City [11], thus obtaining the trajectory sources and proportions of air masses in Dongying City [12]. Each transmission path and direction indicates the regions traversed by the airflow before reaching the receptor point. The transmission distance can determine the airflow transmission speed, and the pressure can determine the height of the air mass [13].

2.6. **Text. S6. PSCF analysis**

PSCF (Potential Source Contribution Function) relies on backward trajectory results and utilizes a series of functions in the TrajStat plugin to calculate WPSCF values. The specific process involves adding observed pollutant concentration data to all trajectories, creating a rectangular grid with a certain resolution in the study area, setting a threshold for the target pollutant, and finally conducting PSCF calculations and weight analysis, followed by visualizing the results into graphics. If the NO_2 concentration corresponding to a trajectory exceeds the set threshold, the trajectory is considered a high-value NO_2 trajectory. The rectangular grid was divided into 0.1°X0.1°, and the PSCF value was the ratio of the number of polluted trajectories within a specific grid to the total number of trajectories passing through that grid. This value describes the probability of the grid point contributing pollutants to the observation point [14]. In this study, the weighted potential source contribution (WPSCF) values were calculated by introducing a weight coefficient W to reduce the error in PSCF values based on conditional probability [15]. The relevant calculation formulas are as follows:

$$WPSCF_{ij} = \frac{m_{ij}}{n_{ij}} \times W_{ij} \tag{1}$$

$$W_{ij} = \begin{cases} 1.00(80 < n_{ij}) \\ 0.70(20 < n_{ij} \leq 80) \\ 0.42(10 < n_{ij} \leq 20) \\ 1.00(n_{ij} \leq 10) \end{cases} \tag{2}$$

In the formula, n_{ij}represents the total number of trajectories passing through grid (i,j), and m_{ij} represents the number of polluted trajectories passing through grid (i,j). The ratio of $n_{ij}$to m_{ij} is the PSCF value. $WPSCF_{ij}$ represents the average weighted contribution probability of grid (i,j).

2.7. **Text. S7. CWT analysis**

The Concentration Weighted Trajectory (CWT) method compensates for the PSCF method's limitation of only assessing pollution probability without determining pollution severity from a concentration perspective [16]. The CWT method is also performed in the TrajStat plugin and does not require setting a pollutant threshold; the rest of the process is similar to the PSCF method. Additionally, this study introduces a weight coefficient W to calculate the Weighted Concentration Weighted Trajectory (WCWT) values. The PSCF formula is as follows:

$$WCWT_{ij} = \frac{\sum_{l=1}^{M} C_l \times \tau_{ijl}}{\sum_{l=1}^{M} \tau_{ijl}} \times W_{ij} \quad (3)$$

l and M represent the trajectory number and the total number of trajectories, respectively. C_l is the mass concentration when trajectory l passes through grid (i,j), τ_{ijl} is the residence time of trajectory l within grid (i,j), and $WCWT_{ij}$ is the average weighted concentration of grid (i,j).

2.8. **Text. S8. Analysis of impact of high NO_2 values on daytime O_3, $PM_{2.5}$, and atmospheric oxidation capacity in Dongying in 2022**

As shown in Fig. S9, compared to NO_2-NHH periods, NO_2-HH events in all seasons lead to significant increases in daytime NO_2, $PM_{2.5}$ and $PM_{2.5}$-bounded NO_3^-, SO_4^{2-} and SOC concentrations. O_3 concentrations increase significantly except in winter. This indicates that NO_2-HH events during nighttime to morning rush hours can affect daytime NO_2 concentrations and promote increases in concentrations of O_3, $PM_{2.5}$ and $PM_{2.5}$-bounded secondary components. In winter, the daytime O_3 concentration during NO_2-HH events is significantly lower than NO_2-NHH periods, possibly due to higher NO concentrations. NO_2-HH events have a greater impact on daytime NO_2, $PM_{2.5}$ and $PM_{2.5}$-bounded secondary components concentrations in autumn and winter, while the impact is smaller in spring and summer. As shown in Fig. S10, during NO_2-HH events across all seasons, O_X concentrations are significantly higher, indicating that NO_2-HH events can enhance the daytime atmospheric oxidation capacity. In spring, autumn, and winter, NOR values increase during NO_2-HH events, whereas in spring and summer, SOR values increase, with no significant changes in autumn and winter. In summary, NO_2-HH events can increase the daytime concentrations of NO_2, O_3, $PM_{2.5}$ and $PM_{2.5}$-bounded secondary components, as well as O_X concentrations, and can also raise NOR and SOR values.

3. Tables

3.1. **Table. S1.**

Table S1. Data types and data sources for each part of analysis in this study

Analysis content	Data type	Data source	Time range
Spatiotemporal distribution characteristics of NO_2 concentration	NO_2 hourly and daily concentration data	Dongying State-controlled and provincial-controlled stations	From January 1, 2017 to December 31, 2023
Impact of NO_2 on air quality levels	NO_2 daily average concentration data and air quality levels data	Dongying State-controlled station	From January 1, 2017 to December 31, 2023
Impact of high values of NO_2 concentration on air quality and the causes of high values of NO_2 concentration in 2022	hourly and daily average concentrations of routine parameters, VOCs, ion components, OC and EC	Dongying Atmospheric Observatory	From January 1 to December 31, 2022
Local emission	NO_X daily average emission data	Dongying Environmental Automatic Monitoring Control System	From January 1, to December 31, 2022
Causes analysis of high NO_2 values	hourly and daily average T, RH, WD and WS	Dongying National Basic Meteorological Station	From January 1, 2017 to December 31, 2022
	hourly BLH		From January 1, to December 31, 2022
	hourly UV	Dongying Atmospheric Observatory	From January 1, to December 31, 2022
Synoptic patterns	ERA5 dataset	European Centre for Medium-Range Weather Forecasts (ECMWF)	From January 1, 2017 to December 31, 2022

3.2. **Table. S2.**

Table S2. Correlation between NO_2 concentration and meteorological factors in different seasons in Dongying from 2017 to 2022.

	Wind speed (WS)	Temperature (T)	Relative humidity (RH)	Atmospheric pressure (AP)	N
Spring	-0.45**	0.02	-0.36**	0.09	552
Summer	-0.18**	0.21**	-0.60**	0.00	537
Autumn	-0.49**	-0.35**	-0.30**	0.29**	545
Winter	-0.57**	0.27**	0.02	-0.24**	552
Year	-0.43**	-0.45**	-0.38**	0.46**	2186

1) Two-tailed significance test: * indicates P<0.05, ** indicates P<0.01
2) N: samples number.

References

1 MEP. Ambient air quality standards (GB 3095-2012). Beijing: China Environmental Science Press. 2012.

2 MEP. Technical Regulation on Ambient Air Quality Index (on Trial) (HJ 633-2012). Beijing: China Environmental Science Press. 2012.

3 MEP. Technical regulation for ambient air quality assessment (on trial) (HJ 663-2013). Beijing: China Environmental Science Press. 2013.

4 Huth, R. A circulation classification scheme applicable in GCM studies. *Theoretical and Applied Climatology*. **2000**, 67, 1-18.

5 Tang, G.Q., LI, X., Wang, X,K., Xin, J,Y., Hu, B., Wang, L.L., Ren, Y.F., Wang, Y.S. Effects of Synoptic Type on Surface Ozone Pollution in Beijing. *Environ. Sci. (China)*. **2010**, 31, 573-578.

6 Liu, Y., Li, Y.h., Hou, X,G., Ma, W. Research on Density and Diffusion Trajectory of NO_2 during Heavy Pollution Period in Urumqi. *Environ. Sci. Technol.* **2017***,* 40, 33-39.

7 Li, Y.J., An, X.Q., Fan, G.Z. Transport pathway and potential source area of atmospheric particulates in Beijing . *China Environ. Sci*. **2019**, 39, 915-927.

8 Wang, Y., Chai, F.H., Wang, Y.H., Liu, M. Transport Characteristics of Air Pollutants over the Yangtze Delta. *Environ. Sci. (China)*. **2008**, 29, 1430-1435.

9 Wang, Y.Q., Zhang, X.Y., Draxler, R.R. TrajStat: GIS-based software that uses various trajectory statistical analysis methods to identify potential sources from long-term air pollution measurement data. *Environ. Model. Softw*. **2009**, 24, 938-939.

10 Xu, H. Temporal and spatial distribution characteristics and pollution sources of NO_2 pollution in Yangtze River Delta. **2022**, Anhui University.

11 Wang, S., Nie, S.S., Feng, Y.P., Cui, J.S., Chem, J., Liu, D.X., Shi, W.Y. Spatio-Temporal Evolution Characteristics and Source Apportionment of O_3 and NO_2 in Shijiazhuang. *Environ. Sci. (China)*. **2021**, 42, 2679-2690.

12 Wang, Z.F., Zhang, W.J., Li, M., Lv, B., Fu, H.X., Sun, F.J., Lv, C.,Bian, M. Analysis of Heavy Air Pollution Episode with Combined Sand Storm and High $PM_{2.5}$ Occurred in Jinan in 2018. *Res. Environ. Sci*. **2021**, 34, 2588-2598.

13 Liu, N., Yu, Y., He, J.J., Zhao, S.P. Analysis of Air Pollutant Transport in Winter in Lanzhou. *Res. Environ. Sci*. **2015**, 28, 509-516.

14 Kulshrestha, U.C., Raman, R.S., Kulshrestha, M.J., Rao, T.N., Hazarika, P.J. Secondary aerosol formation and identification of regional source locations by PSCF analysis in the Indo-Gangetic region of India. *J. Atmos. Chem*. **2009**, 63, 33-47.

15 Hong, Q.Q., Liu, C., Hu, Q.H., Xing, C.Z., Tan, W., Liu, H.R., Huang, Y., Zhu, Y., Zhang, J.S., Geng, T.Z., Liu, J.G. Evolution of the vertical structure of air pollutants during winter heavy pollution episodes: The role of regional transport and potential sources. *Atmos. Res.* **2019**, 228, 206-222.

16 Xu, X., Akhtar, U.S. Identification of potential regional sources of atmospheric total gaseous mercury in Windsor, Ontario, Canada using hybrid receptor modeling. *Atmos. Chem. Phys*. **2010**, 10, 7073-7083.
